# A finite element model of the shoulder: application to the changes of biomechanical environment induced by postoperative malrotation of humeral shaft fracture

**DOI:** 10.1186/s12891-022-05479-3

**Published:** 2022-06-02

**Authors:** Cheng Wang, Xiao-yuan Ma, Lin-tao Lu, Zheng Guo, Guo-feng Dai

**Affiliations:** Department of Orthopaedics, Qilu Hospital of Shandong University, No.107 Wenhua West Road, Jinan, 250012 Shandong Province China

**Keywords:** Finite element model, Biomechanical environment, Postoperative malrotation, Humeral shaft fracture

## Abstract

**Objectives:**

The humerus fracture is one of the most commonly occurring fractures. In this research, we attempted to evaluate and compare the extent of malrotation and biomechanical environment after surgical treatment of humeral shaft fractures.

**Methods:**

A finite element (FE) model of the shoulder was built based on Computed Tomography (CT) data of a patient with a humeral shaft fracture. The muscle group around the shoulder joint was simulated by spring elements. The changes of shoulder stresses under rotation were analyzed. The biomechanics of the normal shoulder and postoperative malrotation of the humeral shaft was analyzed and compared.

**Results:**

During rotations, the maximum stress was centered in the posterosuperior part of the glenoid for the normal shoulder. The von Mises shear stresses were 4.40 MPa and 4.89 MPa at 40° of internal and external rotations, respectively. For internal rotation deformity, the shear contact forces were 7–9 times higher for the shoulder internally rotated 40° than for the normal one. For external rotation deformity, the shear contact forces were about 3–5 times higher for the shoulder with 40° external rotation than the normal one.

**Conclusion:**

Postoperative malrotation of humeral shaft fracture induced the changes of the biomechanical environment of the shoulders. The peak degree of malrotation was correlated with increased stresses of shoulders, which could be paid attention to in humeral shaft fracture treatment. We hoped to provide information about the biomechanical environment of humeral malrotation.

**Supplementary Information:**

The online version contains supplementary material available at 10.1186/s12891-022-05479-3.

## Background

The humerus fracture is one of the most commonly occurring fractures, accounting for 5–8% of all extremity fractures [1], among which shaft fracture is a common long-bone fracture with about 3% incidence [2]. It is estimated that approximately 13 per 100,000 persons are affected by humeral shaft fracture annually. The current management strategies for humerus fracture are multiple, mainly including nonoperative management and surgical treatment such as intramedullary nail (IMN), open reduction internal fixation (ORIF) and minimally invasive plate osteosynthesis (MIPO) technique. In recent years the concept of MIPO being mini-invasive and rendering the shoulder intact seems to be notably popular, however, the postoperative deformity resulting from malreduction using the minimally invasive technique did not receive adequate attention, particularly for individuals with high-level requirements for sports, work or daily life. Despite the advantages of operative management in stability and recovery, the postoperative malrotation of humeral shaft fracture has been a problem to be solved. It is reported that closed nailing could affect retroversion angles and external rotation of the shoulders [3]. The study of Flury et al*.* [4] showed that the more than 20° internal rotation of the humeral head was detected in the patients with post-Weber's rotation osteotomy with shoulder prosthesis. Li et al*.* [5] prospectively analyzed 45 cases with humeral shaft fracture and found that 27.2% of patients who underwent IMN were present with 20° or more internal malrotation of the humeral head. And We also reported even a much higher incidence of 40.9% in the MIPO group [6]. Defects in muscle strength, changes in range of motion (ROM) and degenerative arthritis of the shoulder have been reported as consequences of postoperative malrotation of humeral shaft fractures.

Fjalestad T et al*.* [7] reported that the loss of external rotation in the neutral position affected 38% of the patients who were treated with braces, and the author attributed this phenomenon to humeral head malrotation. Our previous research found a linear correlation between postoperative malrotation and range of rotation loss [8]. Flury et al*.* [4] reported that greater than 20° of postoperative malrotation of the humerus was associated with secondary shoulder arthritis. This finding was mainly attributed to impingement between the malrotated humeral head and the glenoid edge as well as to the increased articular contact stress. As Zaid reviewed, numerous studies confirmed that anatomy parameters of the shoulder, as measured by acromial index (AI), critical shoulder angle (CSA), lateral acromial angle (LAA), and glenoid inclination (GI), appeared to be significantly associated with glenohumeral osteoarthritis [9]. Therefore, we have reason to speculate about the potential impact of humeral head retroversion angle (HRA) change with postoperative malrotation on the shoulder joint.

We became interested in postoperative malrotation of humeral shaft fractures when a typical case was found in our clinical practice and which had been reported in our previous research [5]: an 18- year-old man sustained a left humeral shaft fracture by arm wrestling. Antegrade nailing was performed; however, degenerative arthritis had developed in the involved shoulder 6 years later. When we studied the case, the malrotation of the injured humerus was more than 50 and the degenerative area on the humeral head was in the same location as Flury et al*.* reported (Supplementary Fig. 1A-D).

Therefore, the peak degree of malrotation may cause excessive tensile stress for the humerus, leading to shoulder cartilage damage and degeneration. We consider it very important to understand the biomechanical environment of humeral malrotation. In this study, we built finite element (FE) model (Supplementary Fig. 2) for the humerus shaft and shoulder joint with malrotation and compared the biomechanics of the normal shoulder and postoperative malrotation of the humeral shaft.

## Materials and methods

### Stereolithography model construction

**Fig. 1:**
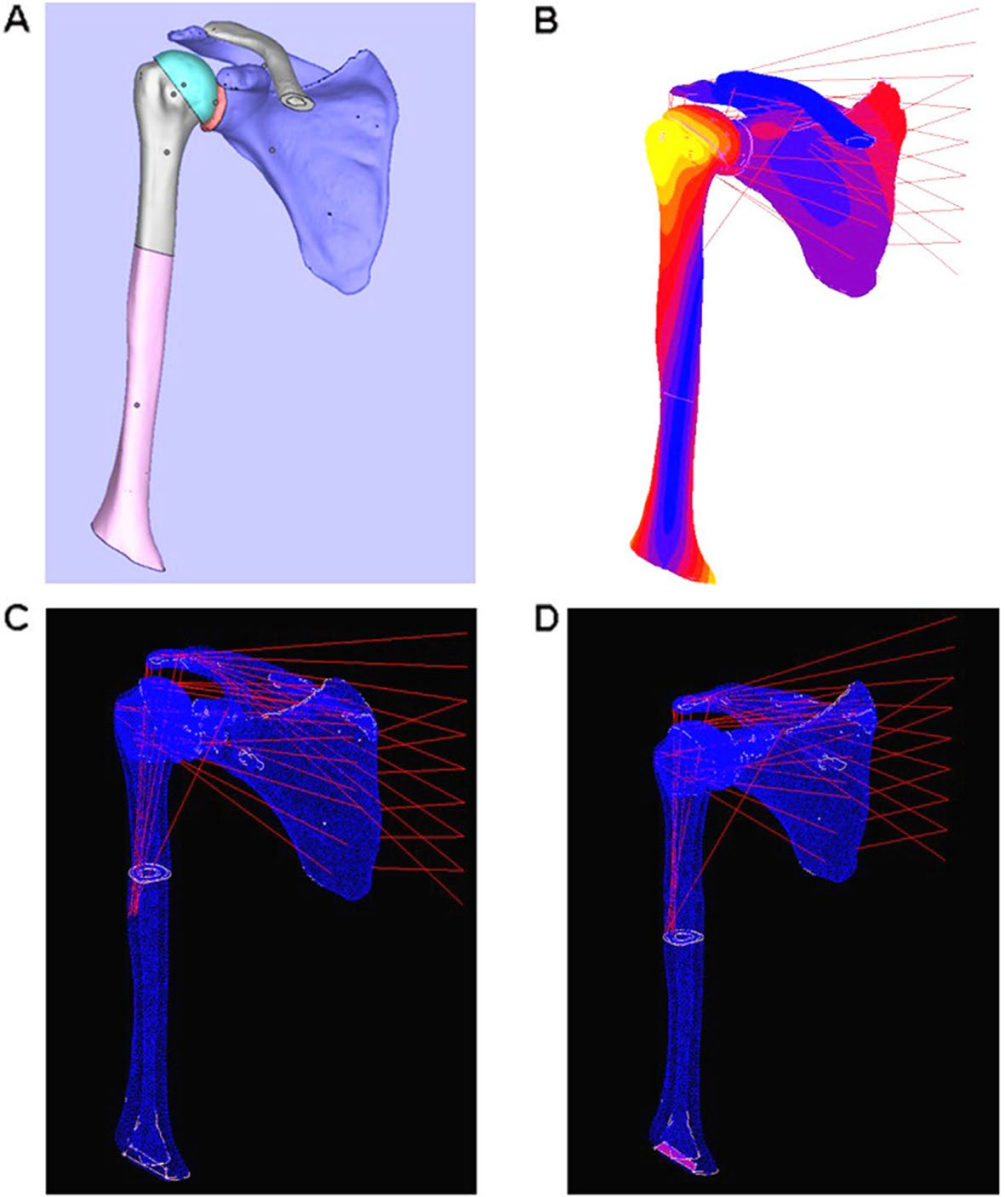
FE model construction. **A** the stereolithography model construction. Based on the CT data of the normal shoulder of a 31-year-old female, the stereolithography model was constructed by MagicRP and Geomagic software. **B** the reconstructed muscles model. The lines represented the spring. **C** postoperative malrotation model of humeral shaft fracture with osteotomy above the insertion of deltoid. **D** postoperative malrotation model of humeral shaft fracture with osteotomy below the insertion of deltoid

This research was approved by the ethical committee of the Medicine College of Shandong University and followed the Declaration of Helsinki and other relevant guidelines and regulations. All experiments and data collection were performed following hospital guidelines. A 31-year-old female patient with a left-side humeral shaft fracture was included in this study and written informed consent was signed by the patient and collected. The bilateral humeral shafts were imaged three times in a supine position by an Aquilion 64 slice spiral CT scanner (Toshiba, Otawara, Japan) with a slice thickness of 1 mm. The CT image data of the right side of humeral shafts (normal shoulder) were collected. Digital imaging and communications in medicine (DICOM) dataset were processed by Mimics 20.0 (Materialise's Interactive Medical Image Control System, Materialise, Belgian). The stereolithography model was constructed by MagicsRP 19.01 and Geomagic studio 2015 (Fig. 1A).

### FE model construction

Based on the stereolithography model, the proximal humerus, scapula and the cartilage in the joint space were meshed by MSC. Marc 2019. Each geometric model meshes into linear tetrahedral elements. Referring to the modeling schemes of Büchler and Zhang [10, 11], material behavior was determined respectively to simulate the bone, cartilage, subchondral bone, muscle and other tissues of the shoulder joint. As described in Table 1, cortical bone and cancellous bone were represented by linear elastic homogeneous, and cartilage was simulated by Neo-Hookean hyperelastic. The muscles were modeled with spring elements, and the number and elastic modulus of springs is determined by the volume, insertion width and function of the muscles. The elastic modulus of springs stimulating muscles to restrain humerus is 50 N/mm, which includes subscapular muscles (6 springs), supraspinatus (3 springs), infraspinatus and teres minor (4 springs), and deltoid (7 springs). The modulus of springs stimulating muscles to stabilize the scapula is 100 N/mm, including trapezius muscle and rhomboid muscle (15 springs). FE model was reconstructed with 258,461 elements and 62,276 nodes (Table 2), and the mesh size was decided following a sensitivity study. For the contact zones, we kept the same size of the elements.

**Table 1:** Description of constitutive laws used in the model. (I_1_ is the first invariant of the Cauchy–Green tensor)

	**Humerus**	**Humeral head cartilage**	**Scapula**	**Glenoid cartilage**	**Cancellus**
Material Behaviour	Linear elastic, Homogeneous	Neo-Hookean Hyperelastic	Linear elastic, Homogeneous	Neo-Hookean Hyperelastic	Linear elastic, Homogeneous
Mathematical expression		W = *C*_10_(I_1_-3)		W = *C*_10_(I_1_-3)	
Constant	E0 = 13.4GPa υ0 = 0.3	*C*_10_ = 1.79	E0 = 9.0GPa υ0 = 0.3	*C*_10_ = 1.79	E0 = 2.0GPa υ0 = 0.2

**Table 2:** Mesh information of finite elements

Import order	Number of units	Number of nodes
The lower section of the humerus	26,930	6119
The upper section of the humerus	45,444	11,160
Articular Cartilage of scapula	19,309	5135
Scapula	51,628	12,837
Clavicle	9856	2342
Cancellus	10,057	2464
Articular Cartilage of proximal humerus head	95,777	22,219

### Loading conditions

The cartesian coordinate system was built with the center of the humeral head as the origin. The Y-axis represented the direction parallel to the axial direction of the humerus bone marrow cavity through the center of the humerus head body. The X-axis was taken as the coronal plane of the cylinder of the medullary cavity with the elbow in front. Z-axis oriented in the direction perpendicular to the coronal plane. There was no change of boundary conditions between normal anatomical structure and post osteotomy geometry.

The glenohumeral contact was defined between two deformable bodies. The material contacts were defined as the contacts between the master (humeral head) and slave (glenoid) surfaces. Two contact rules are defined on the contact surface, in which the normal contact rule is defined as the exponential penetration relationship, which allows penetration from the node on the slave surface to the master surface; Tangential contact law is defined as Coulomb friction law and friction coefficient set asμ = 0. 001. The muscle was modeled with spring elements which are oriented consistent with the direction of muscles contraction. The number of spring elements used was determined based on the ratio of the width and volume of the muscles [11], including the muscles to restraint humerus (20 springs, k = 50 N/mm) and muscles to stabilize the scapula (15 springs, k = 100 N/mm). Figure 1B illustrated the reconstructed muscles modeled with spring elements (Fig. 1B). And the contacts between bone and muscle were considered without friction.

### FE analysis

The mechanical environment of the shoulder joint was analyzed by Marc software in six different conditions. When the elbows were facing straight forward, the shoulder joint was defined as the neutral position (0°). The stress changes under the normal anatomy of the shoulder joint were calculated. Then, stress analysis was performed at 20° internal rotation or external rotation of the proximal humerus and 0–40° internal rotation or external rotation of the shoulder joint for the modeled postoperative malrotation of humeral shaft fracture (Fig. 1C and D).

Considering the mobility of the shoulder joint in daily life and the type and degree of rotation deformity commonly seen in clinical practice, a total of 6 examples were used for FE analysis, including.

I. shoulder joint with 0–40° internal rotation under normal anatomy;
II. shoulder joint with 0–40° external rotation under normal anatomy;
III. osteotomy above deltoid insertion, internal rotation 20° of the proximal humerus and 0–40° internal rotation of shoulder joint;
IV. osteotomy below deltoid insertion, internal rotation 20° of the proximal humerus and 0–40° internal rotation of shoulder joint;
V. osteotomy above deltoid insertion, external rotation 20° of the proximal humerus and 0–40° internal rotation of shoulder joint;
VI. osteotomy above deltoid insertion, external rotation 20° of the proximal humerus and 0–40° external rotation of shoulder joint;

## Results

### Biomechanical analysis of normal shoulder

**Fig. 2:**
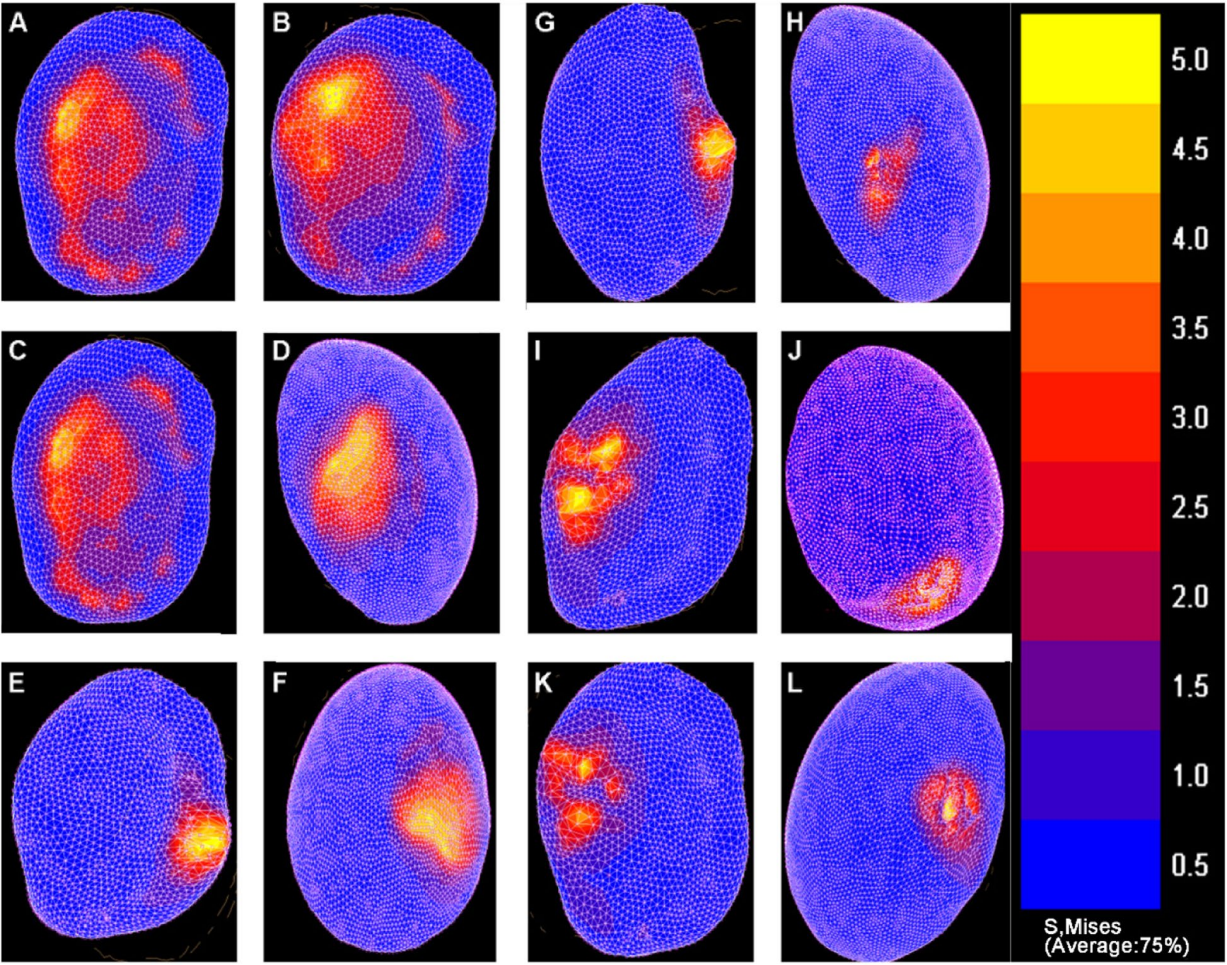
The stress distribution was affected by postoperative malrotation of humeral shaft fracture. The right side of all pictures is anterior and the left is posterior. For normal shoulders, the stress distribution on the articular surface of the glenoid cavity after internal rotation 40° (**A**) and external rotation 40° (**B**). For normal shoulders, the stress distribution on the articular surface of the glenoid cavity (**C**) and humerus head (**D**). Under osteotomy above deltoid insertion, the stress distribution on the articular surface of the glenoid cavity (**E**) and humerus head (**F**) with 20° internal rotation of the proximal humerus and 40° external rotation of the shoulder joint. Under osteotomy below deltoid insertion, the stress distribution on the articular surface of the glenoid cavity (**G**) and humerus head (**H**) with 20° internal rotation of the proximal humerus and 40° external rotation of the shoulder. Under osteotomy above deltoid insertion, the stress distribution on the articular surface of the glenoid cavity (**I**) and humerus head (**J**) with 20° external rotation of the proximal humerus and 40° external rotation of the scapula. Under osteotomy below deltoid insertion, the stress distribution on the articular surface of the glenoid cavity (**K**) and humerus head (**L**) with 20° external rotation of the proximal humerus and 40° external rotation of the scapula

The normal shoulder FE model was built for the biomechanical analysis. Results showed that the glenoid contact pressure was increased with the progressive external and internal rotations of the should joint. During rotations, the maximum stress was centered in the posterosuperior part of the glenoid for the normal shoulder (Fig. 2A-D). The von Mises shear stress was presented as 4.40 MPa and 4.89 MPa at 40° of internal and external rotations, respectively.

### Biomechanical analysis of shoulders with proximal humerus internal rotation deformity

The internal rotation deformity of the proximal humerus model was simulated by osteotomy above the deltoid insertion, and internal rotation 20° of the proximal humerus. Within a range of 0 to 40° internal rotation, the stresses in the shoulder joint were increasing and the stress zone gradually moved to the anteroinferior glenoid fossa of the shoulder (Fig. 2E and F). The maximum von Mises stress was found to be 30.70 MPa for 40° internal rotation.

When the internal rotation deformity of the proximal humerus model was reconstructed by osteotomy below the deltoid insertion, and internal rotation 20° of the proximal humerus. The stress in the glenoid was gradually increasing and the stress concentration zone moved towards the anteroinferior glenoid cavity (Fig. 2G and H). The von Mises stress was calculated to be 37.33 MPa for the maximum internal rotation. Thus, for internal rotation, the shear contact forces were 7–9 times higher for the shoulder internally rotated 40° than for the normal one.

### Biomechanical changes of shoulders with proximal humerus external rotation deformity

To simulate the external rotation deformity of the proximal humerus, osteotomy above or below the insertion of the deltoid was performed, followed by 20° external rotation of the proximal humerus. When the external rotation angle of the shoulder ranged from 0 to 40°, the stress in the glenoid gradually increased and the stress concentration zone was moved to the posterosuperior glenoid cavity (Fig. 2I-L). The maximum von Mises stresses at 40° external of shoulder joint were 16.12 MPa and 24.73 MPa for osteotomy above and below the insertion of deltoid, respectively. These results indicated that for external rotation deformity, the shear contact forces were about 3–5 times higher for the shoulder with 40° external rotation than the normal one.

## Discussion

Humeral shaft fracture is a common injury diagnosed in orthopedics clinics. The postoperative malrotation of the humeral head was closely correlated with surgery outcomes [5]. Besides, the stress distribution of the shoulder joint is different during different motion states, which is dependent on the traction force of surrounding muscles and ligaments. The shoulder degeneration is caused by joint instability [12] and increased stress [13]. Thus, clarifying the stress distribution of the joint shoulder may aid in improving postoperative outcomes.

The stable shoulder was a socket joint with a constant center of rotation [14]. In 1976, Poppen and Walker raised that the soft tissues stabilized the shoulder joint on a relatively fixed center of rotation based on dynamic constraints and mechanical blocking [15]. The study of von et al*.* suggested that the rotation center of the normal shoulder was constant during motion, while in osteoarthritis patients, the rotation center of the shoulder was shifted more than 15 mm [16]. All these indicated that the shoulder joint with degenerative disease was associated with joint instability.

Studies on the stress of shoulder joints were limited in analyzing the anatomy of cadavers in the last century. Warner et al*.* [17] placed the cadavers' normal shoulders on a stress testing apparatus and suggested that the articular contact of the glenohumeral joint was increased after shoulder abduction. Apreleva et al*.* [18] performed experimental investigations on cadavers' normal shoulders to analyze the changes of reaction forces at the glenohumeral joint during shoulder abduction by simulating muscle stretching, which determined the significant role of shoulder biomechanics during active abduction. Furthermore, Bouaicha et al*.* reported that the components of humeral head coverage, glenoid inclination and acromio-glenoidal height showed independent effects on the joint stability and joint reaction forces in a dynamic cadaveric shoulder model [19]. However, the previous studies mentioned above are limited by the complex measurement method, and the inability of stimulating the shoulder motion in a real situation. Currently, little is known about the effect of the postoperative anatomic abnormalities of humerus fracture on the stress distribution of the shoulder.

With the development of mathematical concepts and computerized technology, advances have been achieved in the biomechanical analysis of shoulder joints. Novotny et al*.* developed a mathematical model to predict the glenohumeral kinematics and found that during the external rotation, the anterior band tension was 218.79 with the contact stress up to 0.49 MPa [20]. Dickerson et al*.* developed a computational musculoskeletal model of the shoulder compared with the experimental electromyographic data, which accurately predicted the muscle force in response to external movements [21].

The currently developed FE model has been widely used for the simulation of the shoulder. Buchler et al*.* applied the FE model of the shoulder to compare the stress distribution between the normal shoulder and osteoarthritic shoulder, indicating that the osteoarthritic shoulder was present with posterior subluxation that conformed to the clinical situation [10]. The above proved the availability of the FE model. Besides, the FE model has been thoroughly applied in constructing a musculoskeletal model for dynamically simulating the shoulder function [22].

Rotation deformity of humeral fracture used to be neglected, while correlated researches are rare. As shoulder joint degeneration caused by rotation deformity has been found in clinical practice. Therefore, this aspect should be paid attention to and studied.

In the present study, we reconstructed the FE model of postoperative malrotation of humeral shaft fracture and analyzed the biomechanical environment of the shoulder under malunion. Compared with the previous study [11], the number of meshes of our model is increased to 30 times, which is verified to have better convergence and accuracy. For the 20° internal rotation of the proximal humerus, the shear contact forces were 7–9 times higher for the shoulder internally rotated 40° than for the normal one. The stress concentration zone was located at the glenoid and anteroinferior part of the humerus head. For external rotation deformity, the shear contact forces were about 3–5 times higher for the shoulder with 40° external rotation than the normal one and the stress concentration area was located at the posterosuperior glenoid. Thus, the postoperative malrotation of humeral shaft fracture induced changes in the biomechanical environment of the shoulders, which could be considered in the humeral fracture treatment.

Zhang et al*.* [11] reported that with the increase of rotation angle, the contact stress and contact force increase gradually and The contact stress at 40° is 1.9762 Mpa and the pressure center is posteriorly displaced on the glenoid. Büchler et al*.* [10] simulated the FEA model of the shoulder and found that the contact stress increased with the shoulder joint externally rotation. Novotny et al*.* [20] reported that the external rotation of the shoulder joint was accompanied by the backward displacement of the humeral head. The results of our model analysis are consistent with the above research results, and the peak stress of the scapular glenoid during external rotation in the state of rotational deformity appears in the posterior and inferior part, which is consistent with the epidemiological characteristics that the main wear of shoulder arthritis occurs in the posterior and inferior part of the scapular glenoid and two-thirds of the upper humeral head [7]. And we also had confirmed through animal experiments that rotational deformity of humeral fracture can lead to degeneration of articular cartilage and rotator cuff, which is consistent with the results of this study [6].

There are some limitations in our research. Due to the complexity of shoulders, the present FE models could not thoroughly simulate the biomechanical characteristics of shoulders in vivo. In this study, we just analyzed the bone structure and major muscle groups around the shoulder joint, but without the simulation of the glenoid pelvis and joint capsule ligaments. More complicated models would certainly provide more detailed databases. However, a more comprehensive model construction needs a better understanding of the in vivo functioning and the interaction of different components of the shoulder. Stability of the shoulder is mainly provided by muscles with a minor contribution from the static stabilizers (capsule, labrum and ligaments), and those static stabilization structures mainly function to assist with stability when the shoulder joint reaches or exceeds the limit of the range of motion. Thus, the analysis deviation caused by the lack of static stabilization structures in our research model may not affect the tendency of the analysis results. The FE model we applied was verified based on previous literature, whereas fully validated shoulder FE models to evaluate shoulder mobility and stability need to be established in the future.

As in many of the previous studies [10, 11, 23, 24], only part of the shoulder complex was considered for model simplification and the boundary and loading conditions of our model were artificially imposed. This model only simulates the restraint and stabilization effect of muscles on the shoulder joint, but cannot simulate the muscles activation. Some researchers [25–27] used data collected from cadaver experiments or roughly estimated muscle forces to define boundary and loading conditions, which possibly provide more profound results. However, despite the complexity of more comprehensive models, the data collected from in vitro experiments or merely roughly estimated may be far from the reality of in vivo biomechanical environment. In the future, advances in medical imaging, subject-specific definition and in vivo force sensing techniques may help to establish a fully validated model capable of describing the realistic physiological conditions of the shoulder complex [28].

Our model is helpful to evaluate the biomechanical environment alteration caused by variation or acquired changes of shoulder bone anatomical parameters. Especially for clinical problems such as humeral shaft fracture, the model could help to evaluate the long-term shoulder function and stability in those patients treated with an intramedullary nail and percutaneous plate fixation. And also for shoulder arthroplasty, the model could be introduced to analyze the correlation of the stability and long-term prosthesis survival with the retroversion angle of humeral prosthesis design or placement.

## Conclusion

A peak degree of malrotation significantly increased the stress of the shoulders. The internal rotation deformity of the proximal humerus could lead to the degenerative disease of the shoulder for the excess stress in the glenoid pelvis and anteroinferior head of the humerus. The external rotation deformity could induce a relatively low degree of stress increase in the shoulders, which might increase the risk of shoulder dislocation. Thus, the postoperative malrotation of humerus shaft fracture should be considered in the follow-up period.

## Supplementary Information

**Additional file 1.**

**Additional file 2.**

**Additional file 3.**

**Additional file 4.**

**Additional file 5.**

**Additional file 6.**

**Additional file 7.**

**Additional file 8.**

## Data Availability

The datasets used during the present study are available from the corresponding author upon reasonable request.

## Abbreviations

FE: A finite element
IMN: Intramedullary nail
ORIF: Open reduction internal fixation
DICOM: The digital imaging and communications in medicine
STL: Standard template library
