# Supplementary figures and images for: A finite element model of the shoulder: application to the changes of biomechanical environment induced by postoperative malrotation of humeral shaft fracture
Source: BMC Musculoskelet Disord. 2022 Jun 2;23:525. doi: 10.1186/s12891-022-05479-3 (PMC9161483; doi:10.1186/s12891-022-05479-3)

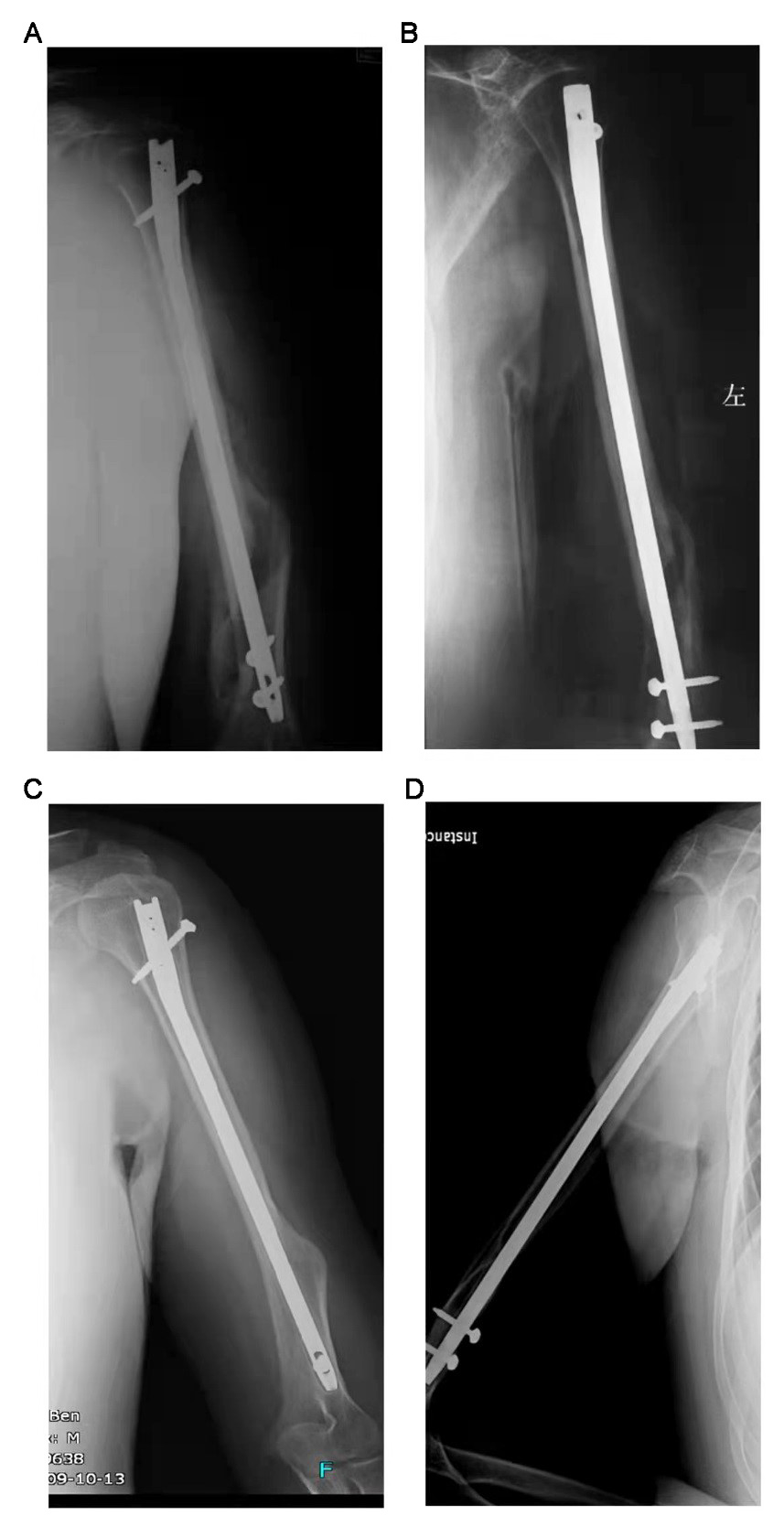

Supplement: Supplementary file 1 — Additional file 1. [file 12891_2022_5479_MOESM1_ESM.tif]

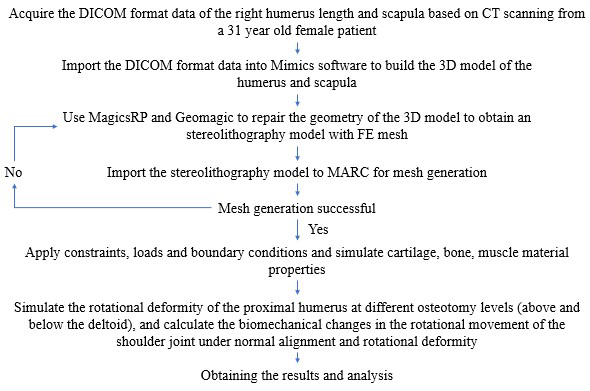

Supplement: Supplementary file 2 — Additional file 2. [file 12891_2022_5479_MOESM2_ESM.tif]

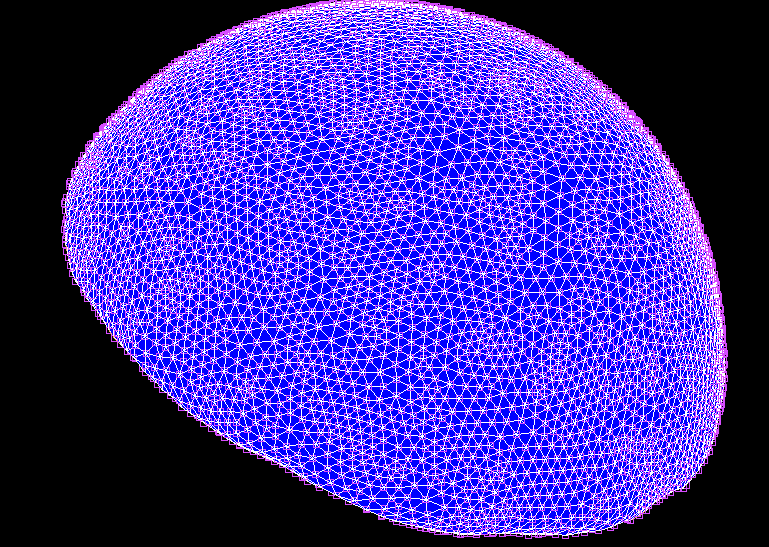

Supplement: Supplementary file 8 — Additional file 8. [file 12891_2022_5479_MOESM8_ESM.zip › Figures/Distal external rotation 20 fracture/External rotation-head/═Γ╨220╢╚╜╙╣╟-═Γ╨2-0╢╚-═╖.png]

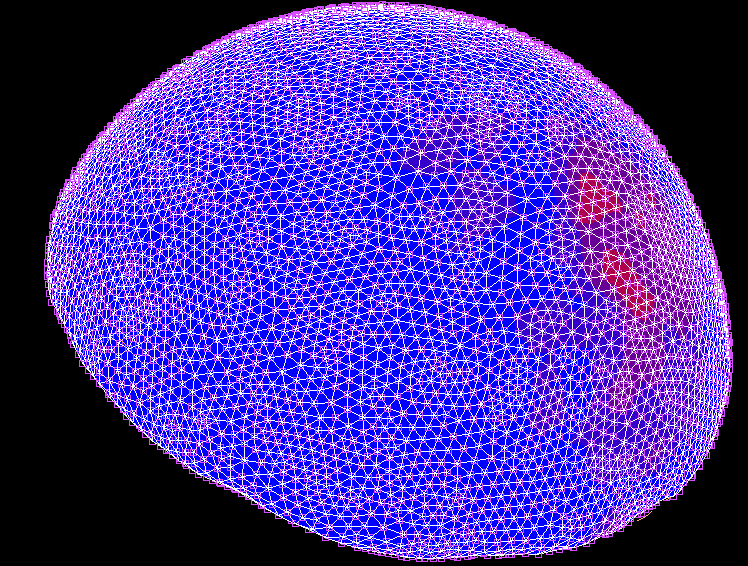

Supplement: Supplementary file 8 — Additional file 8. [file 12891_2022_5479_MOESM8_ESM.zip › Figures/Distal external rotation 20 fracture/External rotation-head/═Γ╨220╢╚╜╙╣╟-═Γ╨2-10╢╚-═╖.png]

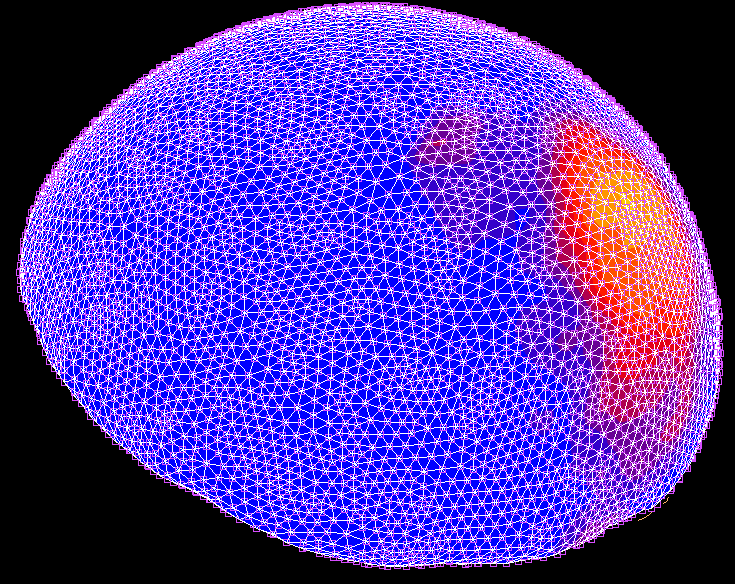

Supplement: Supplementary file 8 — Additional file 8. [file 12891_2022_5479_MOESM8_ESM.zip › Figures/Distal external rotation 20 fracture/External rotation-head/═Γ╨220╢╚╜╙╣╟-═Γ╨2-20╢╚-═╖.png]

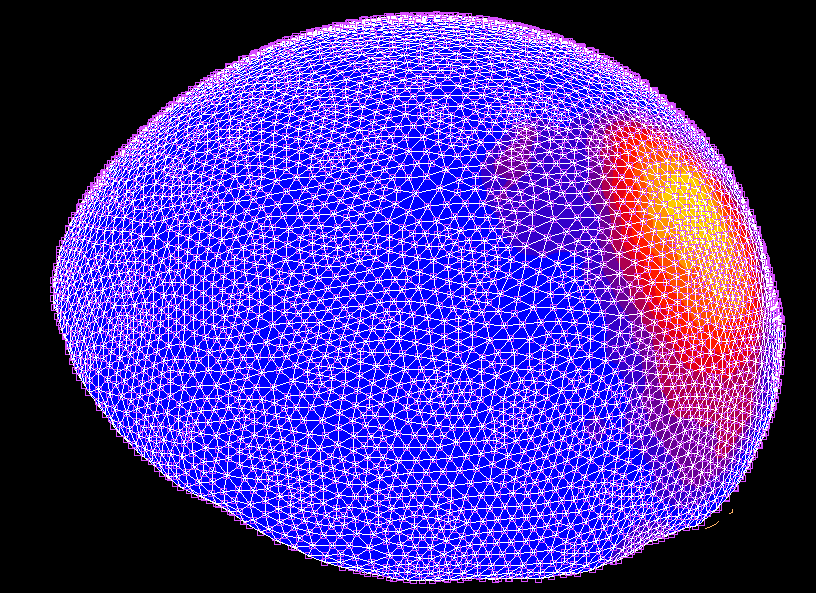

Supplement: Supplementary file 8 — Additional file 8. [file 12891_2022_5479_MOESM8_ESM.zip › Figures/Distal external rotation 20 fracture/External rotation-head/═Γ╨220╢╚╜╙╣╟-═Γ╨2-30╢╚-═╖.png]

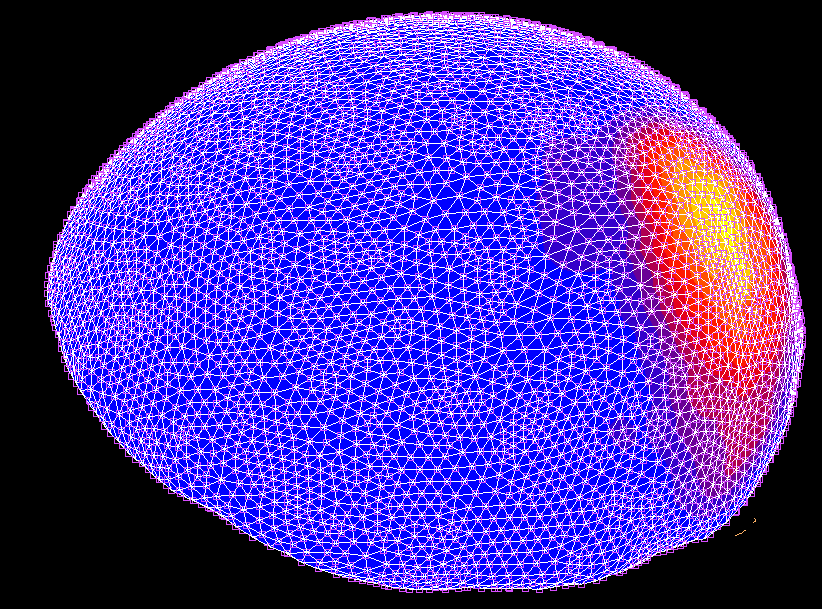

Supplement: Supplementary file 8 — Additional file 8. [file 12891_2022_5479_MOESM8_ESM.zip › Figures/Distal external rotation 20 fracture/External rotation-head/═Γ╨220╢╚╜╙╣╟-═Γ╨2-40╢╚-═╖.png]

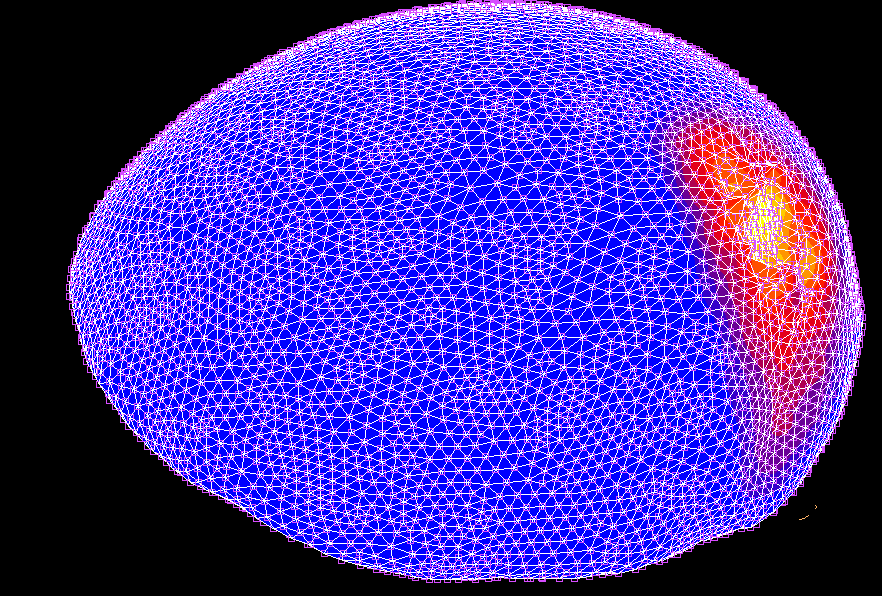

Supplement: Supplementary file 8 — Additional file 8. [file 12891_2022_5479_MOESM8_ESM.zip › Figures/Distal external rotation 20 fracture/External rotation-head/═Γ╨220╢╚╜╙╣╟-═Γ╨2-50╢╚-═╖.png]

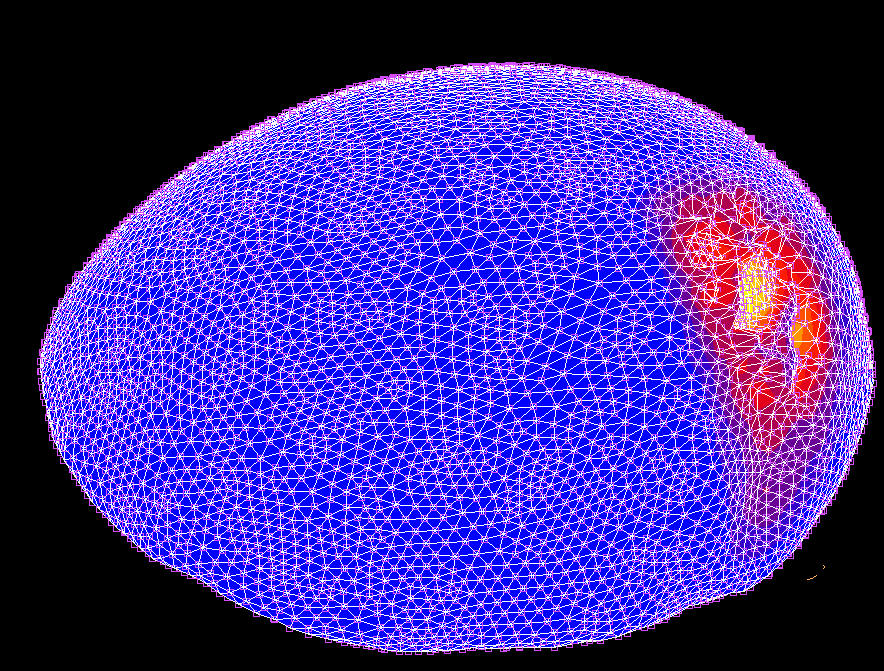

Supplement: Supplementary file 8 — Additional file 8. [file 12891_2022_5479_MOESM8_ESM.zip › Figures/Distal external rotation 20 fracture/External rotation-head/═Γ╨220╢╚╜╙╣╟-═Γ╨2-60╢╚-═╖.png]

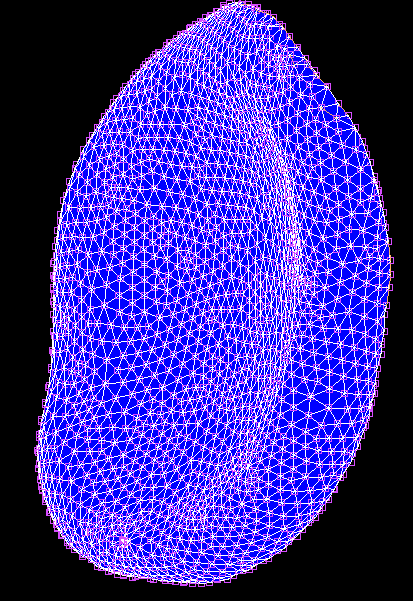

Supplement: Supplementary file 8 — Additional file 8. [file 12891_2022_5479_MOESM8_ESM.zip › Figures/Distal external rotation 20 fracture/External rotation-scapular/═Γ╨220╢╚╜╙╣╟-─┌╨20╢╚.png]

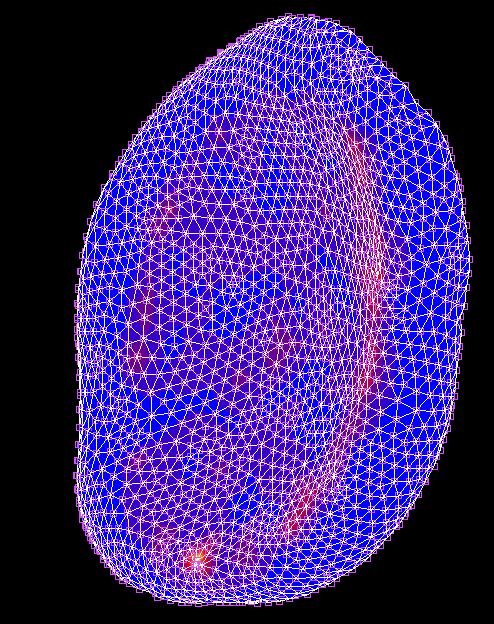

Supplement: Supplementary file 8 — Additional file 8. [file 12891_2022_5479_MOESM8_ESM.zip › Figures/Distal external rotation 20 fracture/External rotation-scapular/═Γ╨220╢╚╜╙╣╟-─┌╨210╢╚.png]

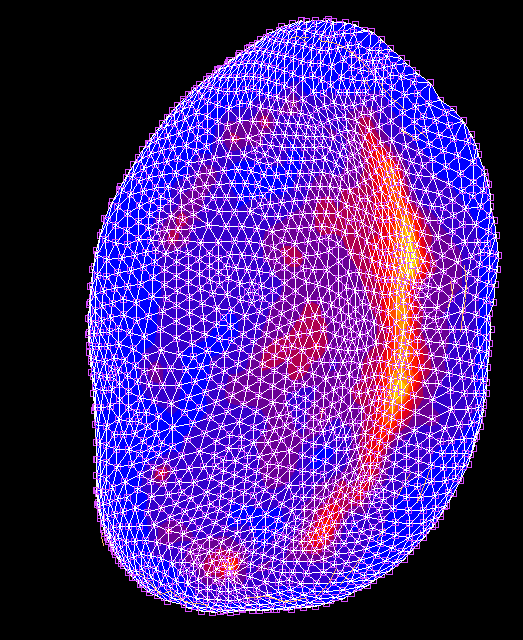

Supplement: Supplementary file 8 — Additional file 8. [file 12891_2022_5479_MOESM8_ESM.zip › Figures/Distal external rotation 20 fracture/External rotation-scapular/═Γ╨220╢╚╜╙╣╟-─┌╨220╢╚.png]

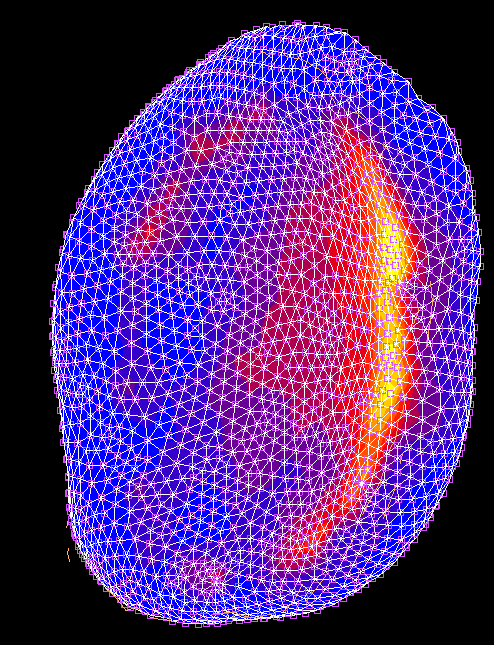

Supplement: Supplementary file 8 — Additional file 8. [file 12891_2022_5479_MOESM8_ESM.zip › Figures/Distal external rotation 20 fracture/External rotation-scapular/═Γ╨220╢╚╜╙╣╟-─┌╨230╢╚.png]

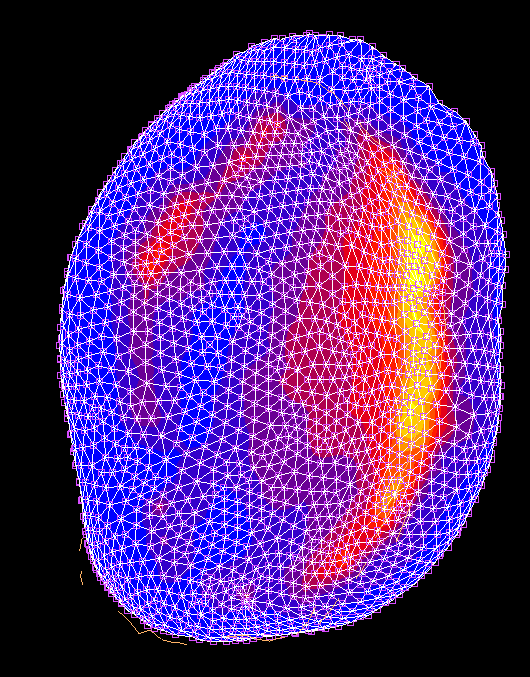

Supplement: Supplementary file 8 — Additional file 8. [file 12891_2022_5479_MOESM8_ESM.zip › Figures/Distal external rotation 20 fracture/External rotation-scapular/═Γ╨220╢╚╜╙╣╟-─┌╨240╢╚.png]

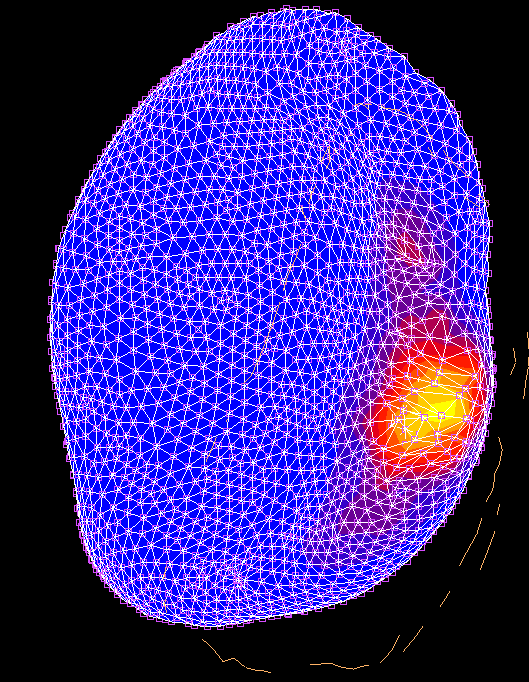

Supplement: Supplementary file 8 — Additional file 8. [file 12891_2022_5479_MOESM8_ESM.zip › Figures/Distal external rotation 20 fracture/External rotation-scapular/═Γ╨220╢╚╜╙╣╟-─┌╨250╢╚.png]

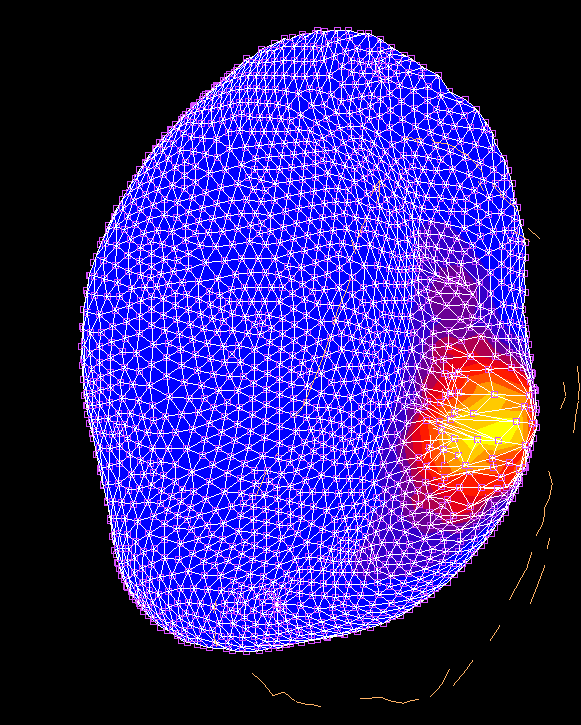

Supplement: Supplementary file 8 — Additional file 8. [file 12891_2022_5479_MOESM8_ESM.zip › Figures/Distal external rotation 20 fracture/External rotation-scapular/═Γ╨220╢╚╜╙╣╟-─┌╨260╢╚.png]

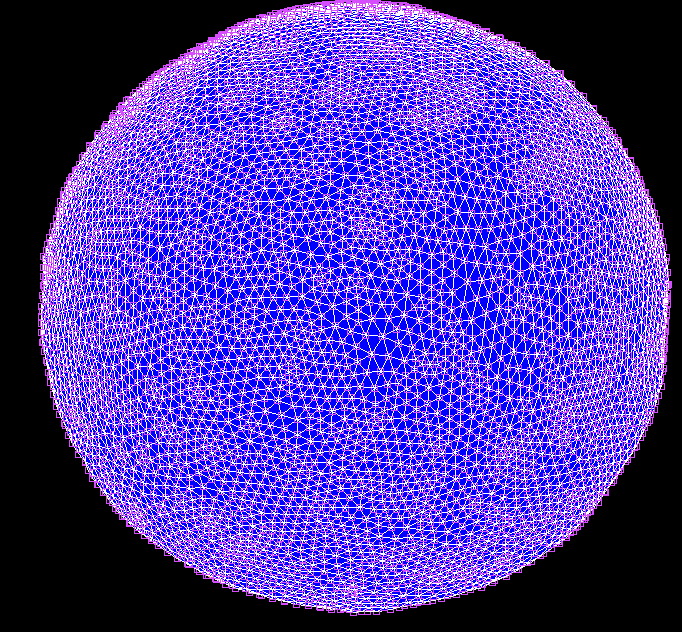

Supplement: Supplementary file 8 — Additional file 8. [file 12891_2022_5479_MOESM8_ESM.zip › Figures/Distal external rotation 20 fracture/Internal rotation-head/═Γ╨220╢╚╜╙╣╟-═Γ╨2-0╢╚-═╖.png]

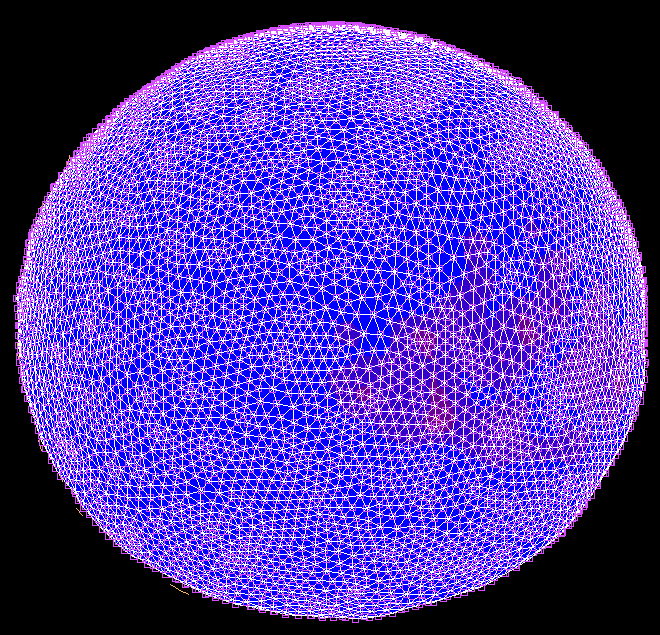

Supplement: Supplementary file 8 — Additional file 8. [file 12891_2022_5479_MOESM8_ESM.zip › Figures/Distal external rotation 20 fracture/Internal rotation-head/═Γ╨220╢╚╜╙╣╟-═Γ╨2-10╢╚-═╖.png]

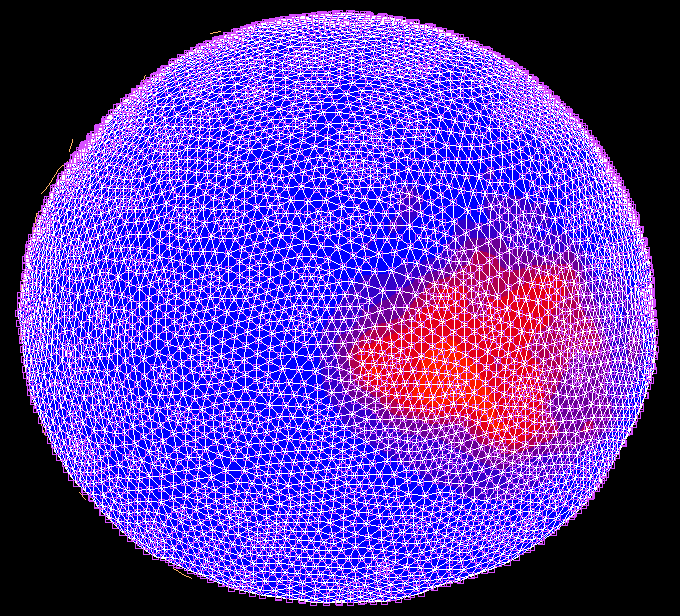

Supplement: Supplementary file 8 — Additional file 8. [file 12891_2022_5479_MOESM8_ESM.zip › Figures/Distal external rotation 20 fracture/Internal rotation-head/═Γ╨220╢╚╜╙╣╟-═Γ╨2-20╢╚-═╖.png]

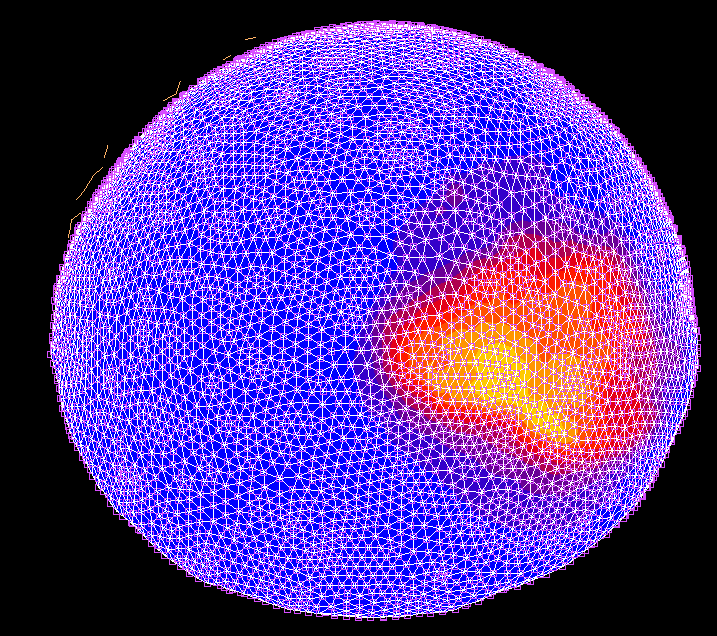

Supplement: Supplementary file 8 — Additional file 8. [file 12891_2022_5479_MOESM8_ESM.zip › Figures/Distal external rotation 20 fracture/Internal rotation-head/═Γ╨220╢╚╜╙╣╟-═Γ╨2-30╢╚-═╖.png]

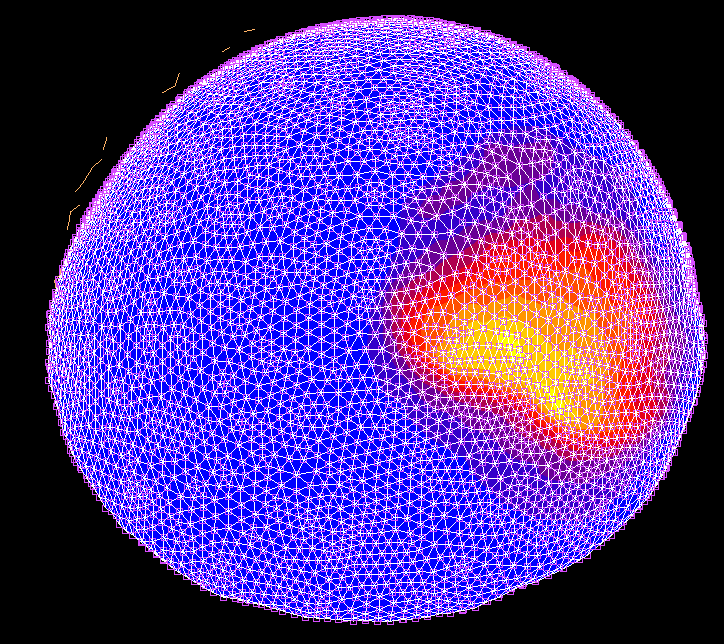

Supplement: Supplementary file 8 — Additional file 8. [file 12891_2022_5479_MOESM8_ESM.zip › Figures/Distal external rotation 20 fracture/Internal rotation-head/═Γ╨220╢╚╜╙╣╟-═Γ╨2-40╢╚-═╖.png]

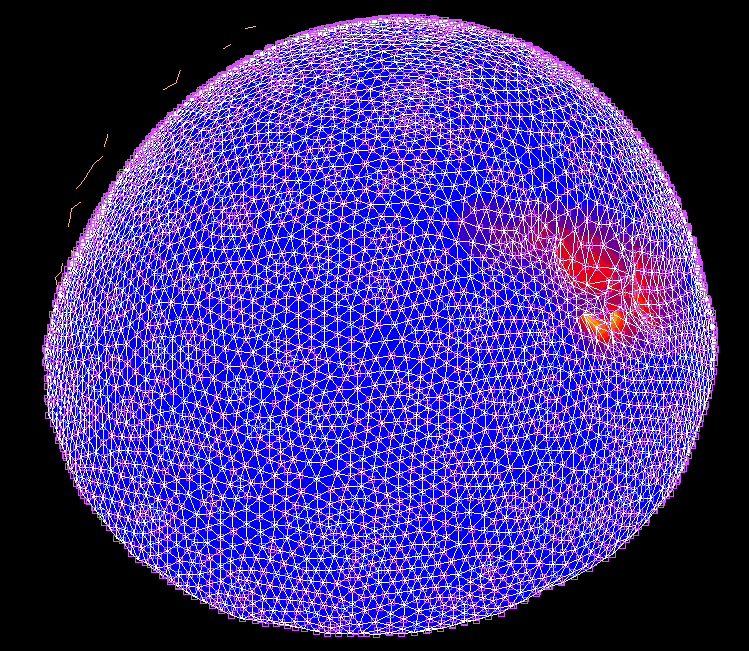

Supplement: Supplementary file 8 — Additional file 8. [file 12891_2022_5479_MOESM8_ESM.zip › Figures/Distal external rotation 20 fracture/Internal rotation-head/═Γ╨220╢╚╜╙╣╟-═Γ╨2-50╢╚-═╖.png]

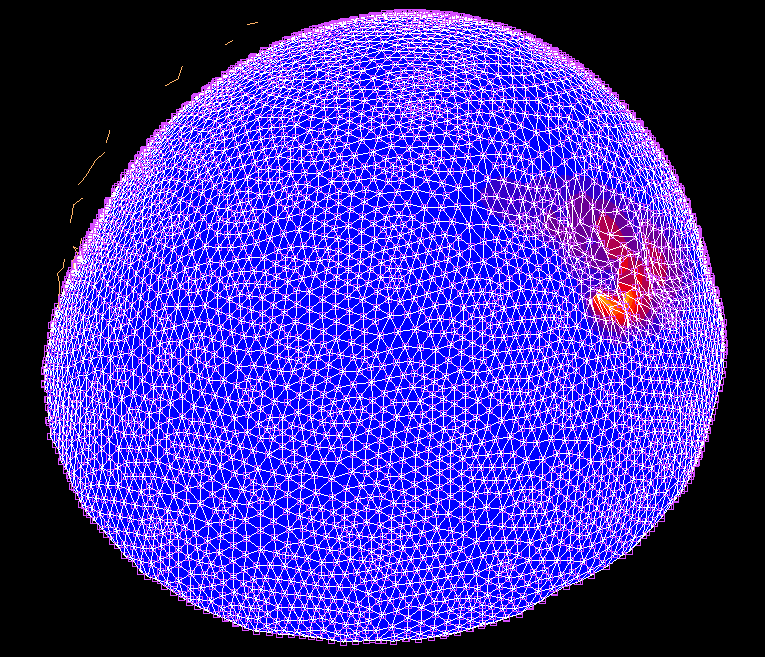

Supplement: Supplementary file 8 — Additional file 8. [file 12891_2022_5479_MOESM8_ESM.zip › Figures/Distal external rotation 20 fracture/Internal rotation-head/═Γ╨220╢╚╜╙╣╟-═Γ╨2-60╢╚-═╖.png]

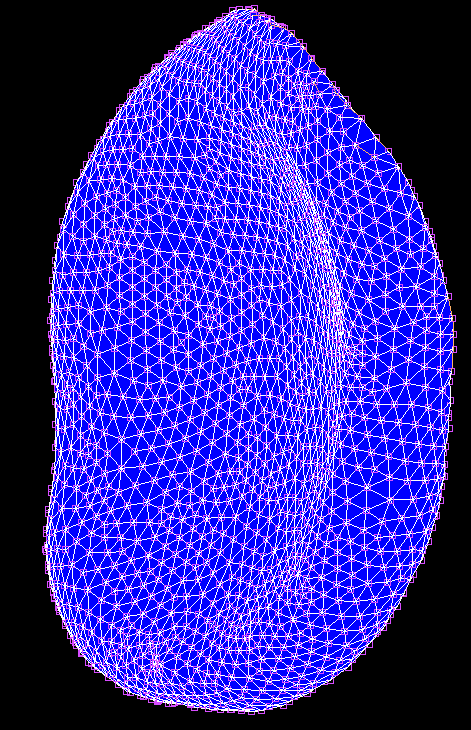

Supplement: Supplementary file 8 — Additional file 8. [file 12891_2022_5479_MOESM8_ESM.zip › Figures/Distal external rotation 20 fracture/Internal rotation-scapular/═Γ╨220╢╚╜╙╣╟-═Γ╨2-0╢╚.png]

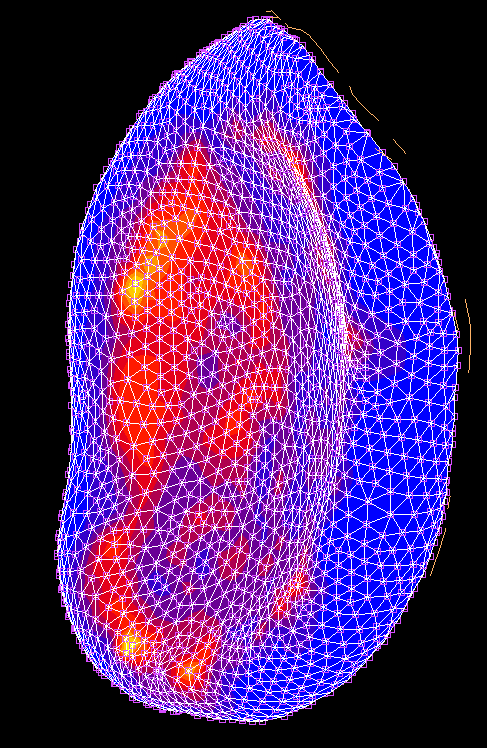

Supplement: Supplementary file 8 — Additional file 8. [file 12891_2022_5479_MOESM8_ESM.zip › Figures/Distal external rotation 20 fracture/Internal rotation-scapular/═Γ╨220╢╚╜╙╣╟-═Γ╨2-10╢╚.png]

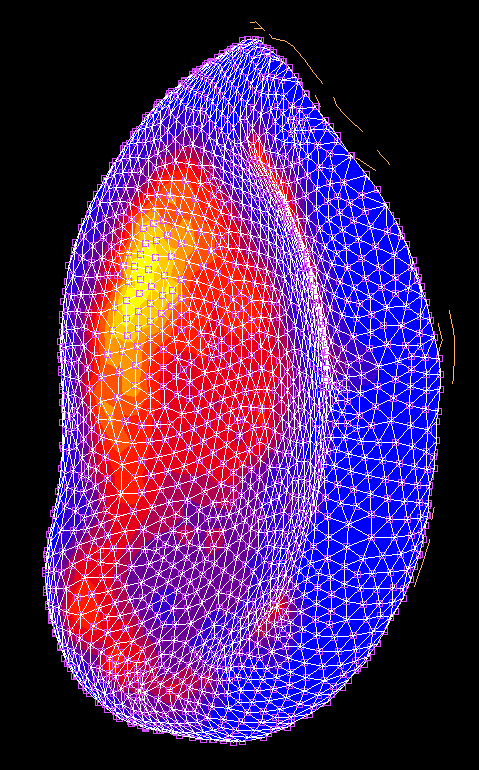

Supplement: Supplementary file 8 — Additional file 8. [file 12891_2022_5479_MOESM8_ESM.zip › Figures/Distal external rotation 20 fracture/Internal rotation-scapular/═Γ╨220╢╚╜╙╣╟-═Γ╨2-20╢╚.png]

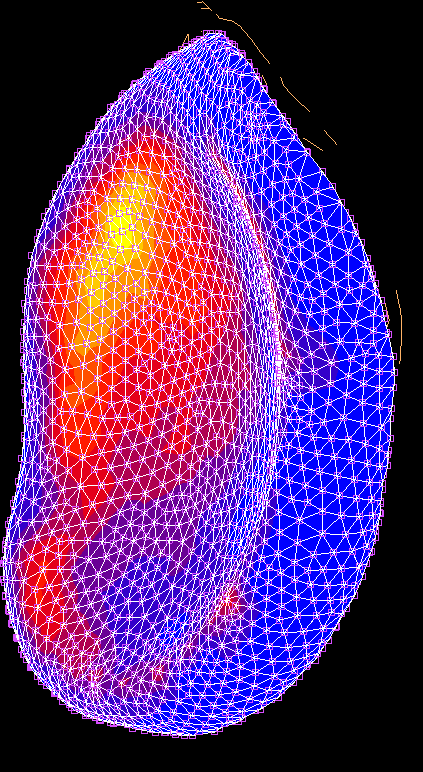

Supplement: Supplementary file 8 — Additional file 8. [file 12891_2022_5479_MOESM8_ESM.zip › Figures/Distal external rotation 20 fracture/Internal rotation-scapular/═Γ╨220╢╚╜╙╣╟-═Γ╨2-30╢╚.png]

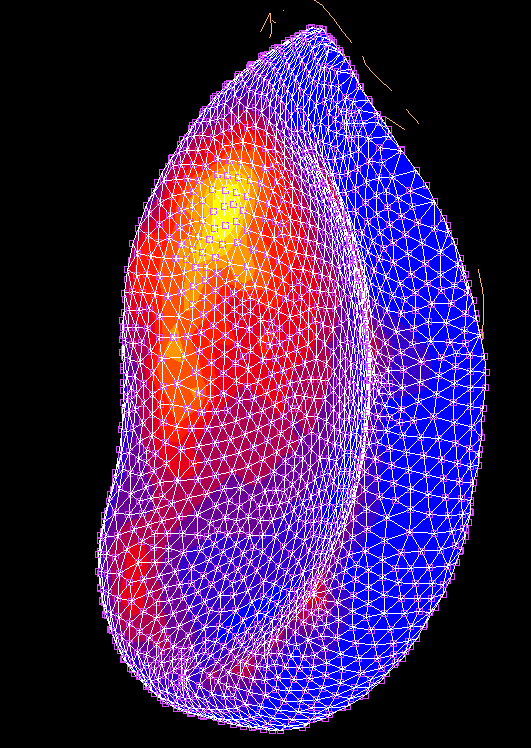

Supplement: Supplementary file 8 — Additional file 8. [file 12891_2022_5479_MOESM8_ESM.zip › Figures/Distal external rotation 20 fracture/Internal rotation-scapular/═Γ╨220╢╚╜╙╣╟-═Γ╨2-40╢╚.png]

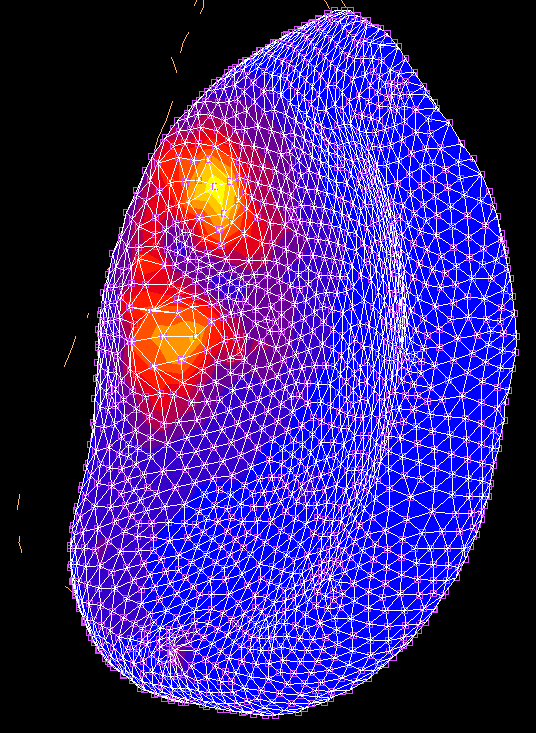

Supplement: Supplementary file 8 — Additional file 8. [file 12891_2022_5479_MOESM8_ESM.zip › Figures/Distal external rotation 20 fracture/Internal rotation-scapular/═Γ╨220╢╚╜╙╣╟-═Γ╨2-50╢╚.png]

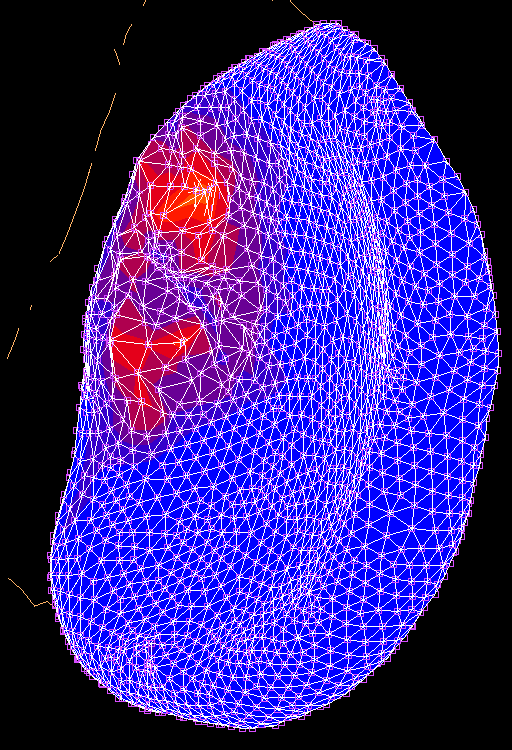

Supplement: Supplementary file 8 — Additional file 8. [file 12891_2022_5479_MOESM8_ESM.zip › Figures/Distal external rotation 20 fracture/Internal rotation-scapular/═Γ╨220╢╚╜╙╣╟-═Γ╨2-60╢╚.png]

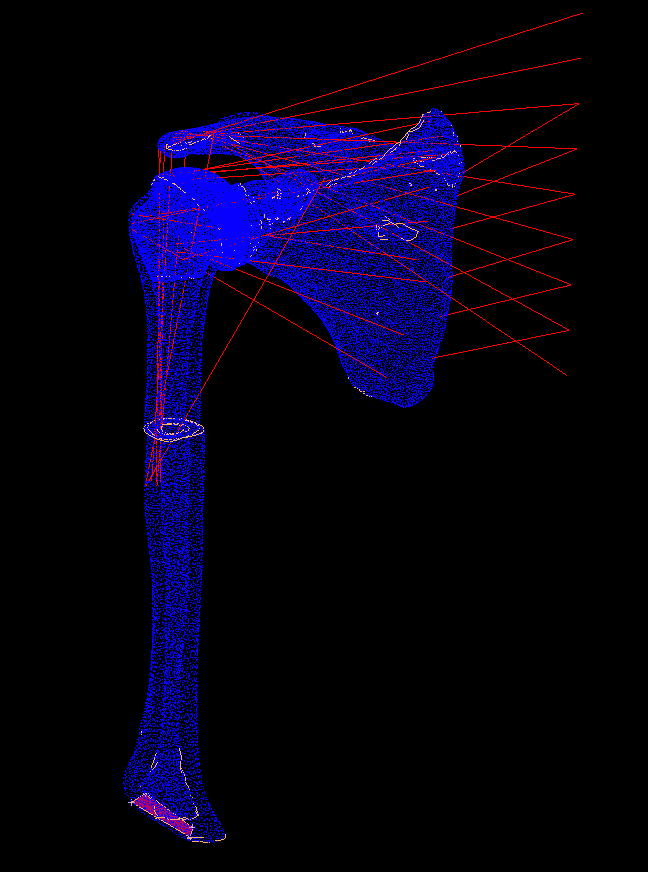

Supplement: Supplementary file 8 — Additional file 8. [file 12891_2022_5479_MOESM8_ESM.zip › Figures/Distal external rotation 20 fracture/Model.png]

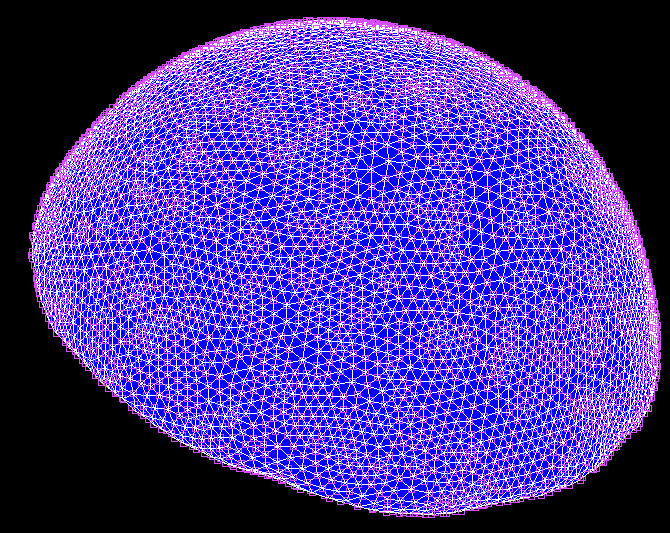

Supplement: Supplementary file 8 — Additional file 8. [file 12891_2022_5479_MOESM8_ESM.zip › Figures/Distal internal rotation 20 fracture/External rotation-head/─┌╨220╢╚╜╙╣╟-═Γ╨2-0╢╚-═╖.png]

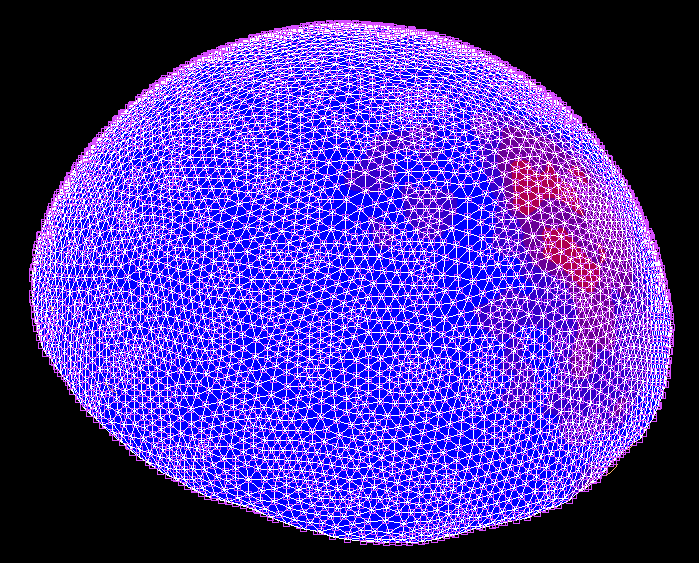

Supplement: Supplementary file 8 — Additional file 8. [file 12891_2022_5479_MOESM8_ESM.zip › Figures/Distal internal rotation 20 fracture/External rotation-head/─┌╨220╢╚╜╙╣╟-═Γ╨2-10╢╚-═╖.png]

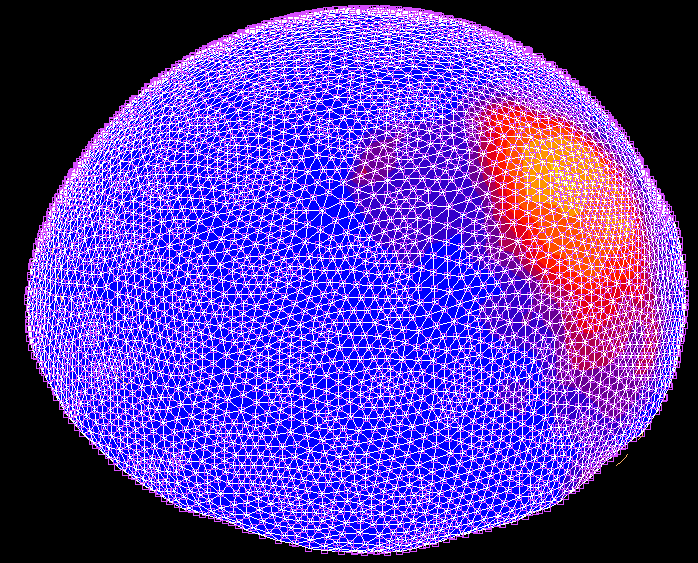

Supplement: Supplementary file 8 — Additional file 8. [file 12891_2022_5479_MOESM8_ESM.zip › Figures/Distal internal rotation 20 fracture/External rotation-head/─┌╨220╢╚╜╙╣╟-═Γ╨2-20╢╚-═╖.png]

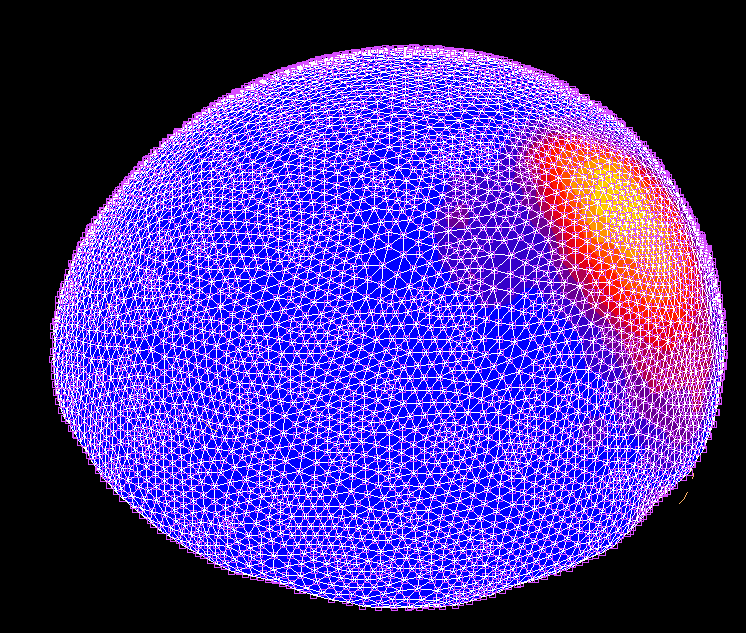

Supplement: Supplementary file 8 — Additional file 8. [file 12891_2022_5479_MOESM8_ESM.zip › Figures/Distal internal rotation 20 fracture/External rotation-head/─┌╨220╢╚╜╙╣╟-═Γ╨2-30╢╚-═╖.png]

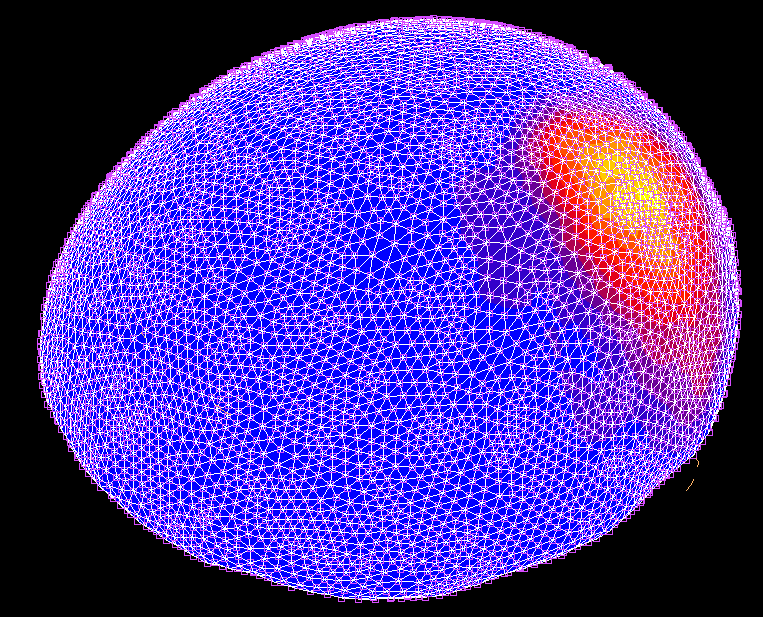

Supplement: Supplementary file 8 — Additional file 8. [file 12891_2022_5479_MOESM8_ESM.zip › Figures/Distal internal rotation 20 fracture/External rotation-head/─┌╨220╢╚╜╙╣╟-═Γ╨2-40╢╚-═╖.png]

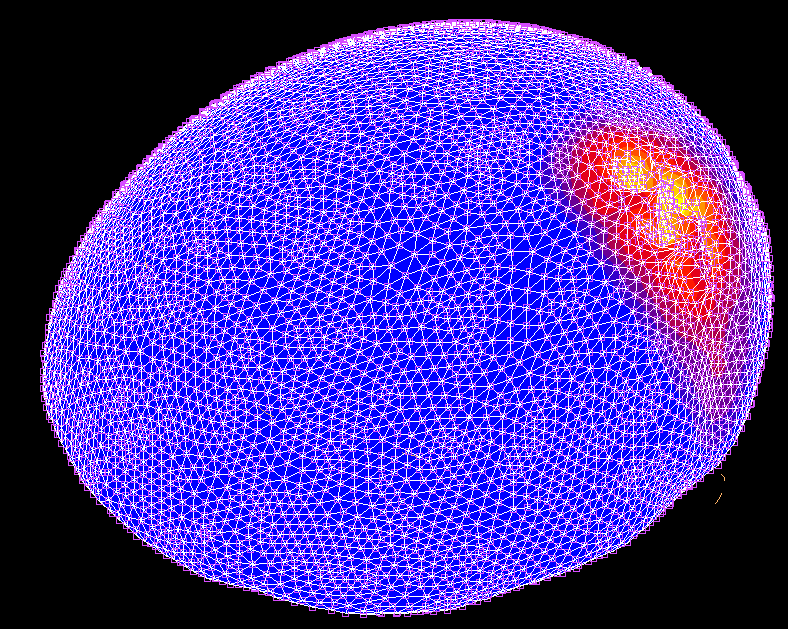

Supplement: Supplementary file 8 — Additional file 8. [file 12891_2022_5479_MOESM8_ESM.zip › Figures/Distal internal rotation 20 fracture/External rotation-head/─┌╨220╢╚╜╙╣╟-═Γ╨2-50╢╚-═╖.png]

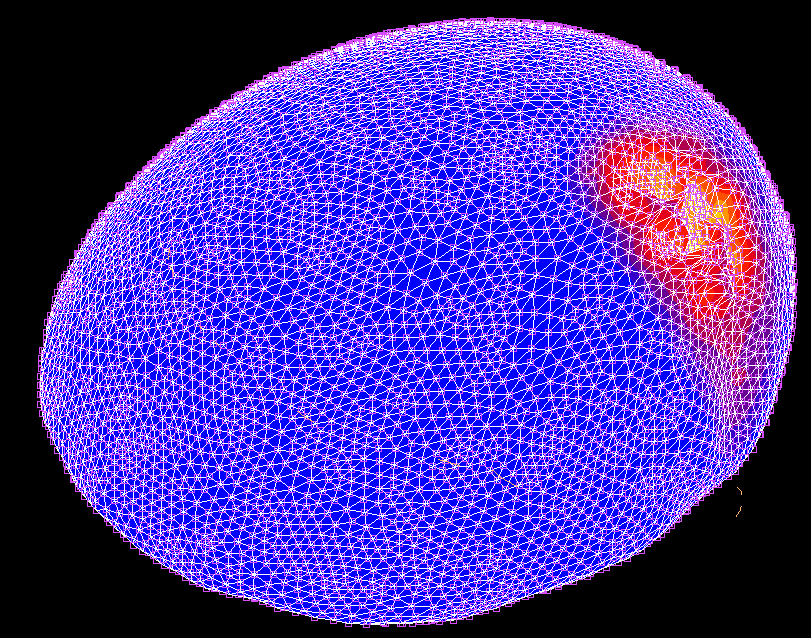

Supplement: Supplementary file 8 — Additional file 8. [file 12891_2022_5479_MOESM8_ESM.zip › Figures/Distal internal rotation 20 fracture/External rotation-head/─┌╨220╢╚╜╙╣╟-═Γ╨2-60╢╚-═╖.png]

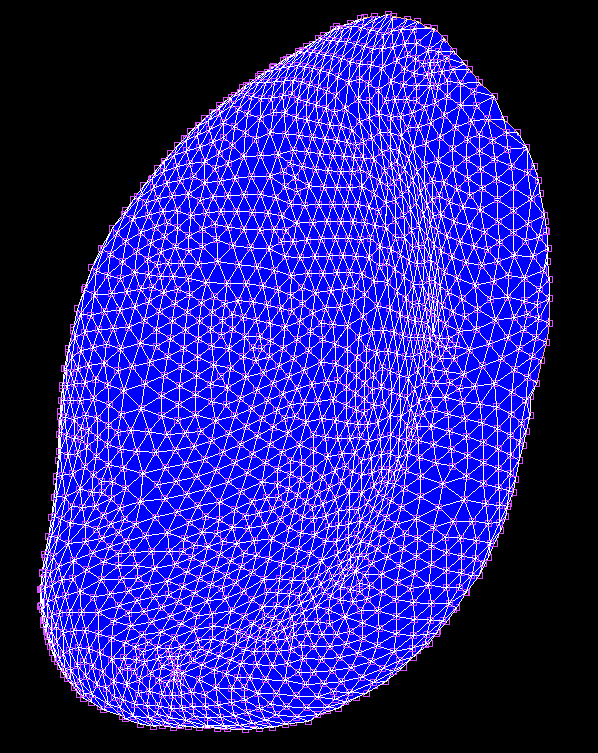

Supplement: Supplementary file 8 — Additional file 8. [file 12891_2022_5479_MOESM8_ESM.zip › Figures/Distal internal rotation 20 fracture/External rotation-scapular/─┌╨220╢╚╜╙╣╟-═Γ╨2-0╢╚.png]

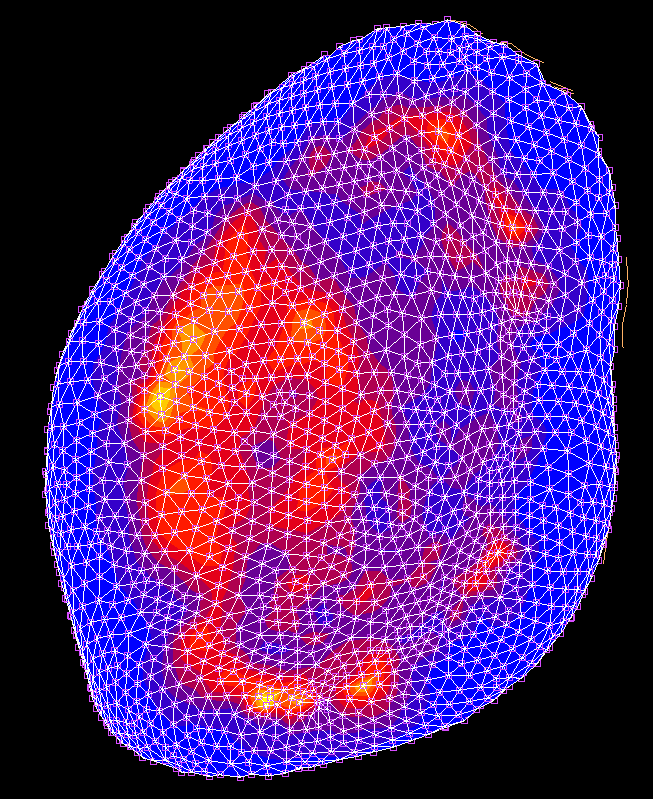

Supplement: Supplementary file 8 — Additional file 8. [file 12891_2022_5479_MOESM8_ESM.zip › Figures/Distal internal rotation 20 fracture/External rotation-scapular/─┌╨220╢╚╜╙╣╟-═Γ╨2-10╢╚.png]

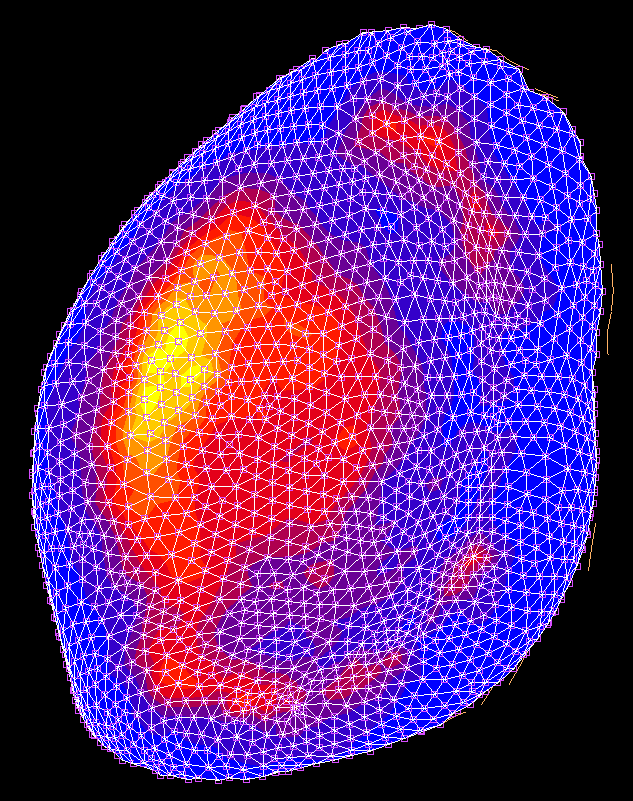

Supplement: Supplementary file 8 — Additional file 8. [file 12891_2022_5479_MOESM8_ESM.zip › Figures/Distal internal rotation 20 fracture/External rotation-scapular/─┌╨220╢╚╜╙╣╟-═Γ╨2-20╢╚.png]

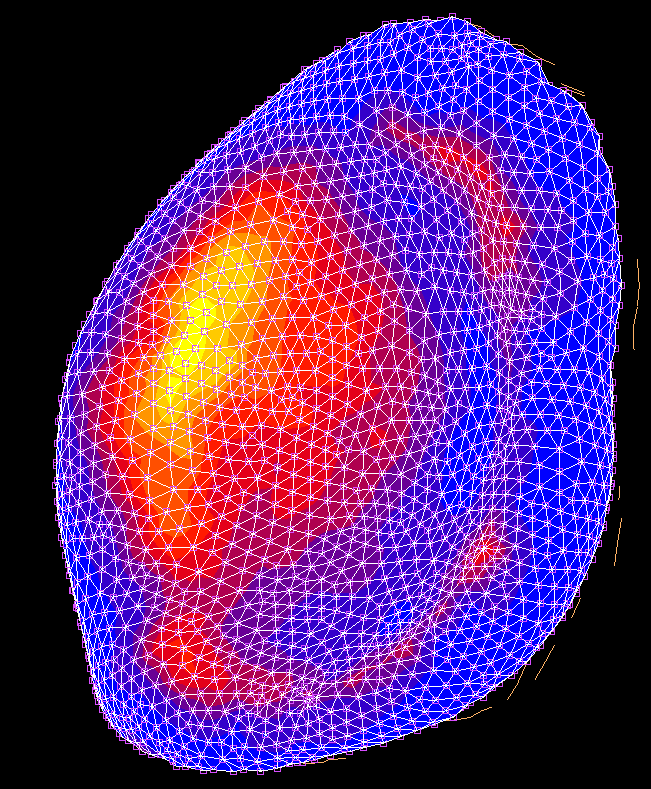

Supplement: Supplementary file 8 — Additional file 8. [file 12891_2022_5479_MOESM8_ESM.zip › Figures/Distal internal rotation 20 fracture/External rotation-scapular/─┌╨220╢╚╜╙╣╟-═Γ╨2-30╢╚.png]

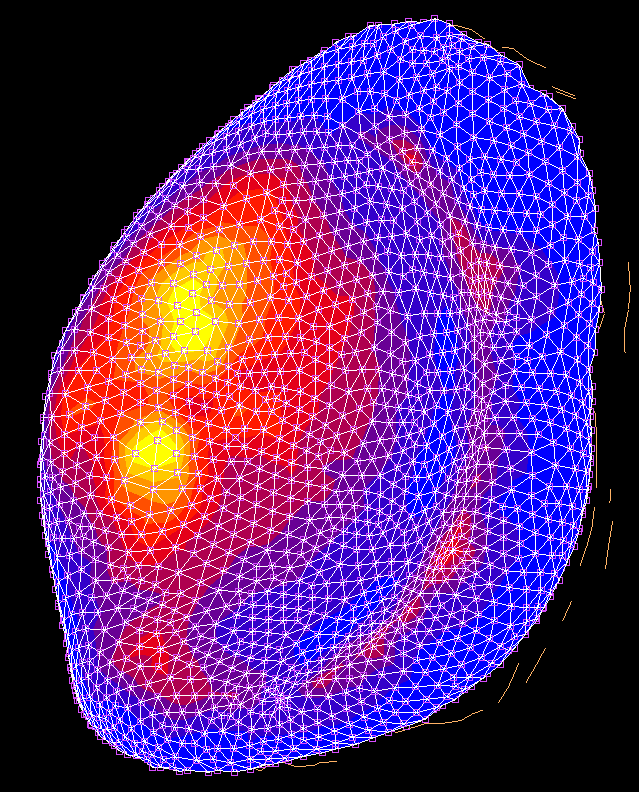

Supplement: Supplementary file 8 — Additional file 8. [file 12891_2022_5479_MOESM8_ESM.zip › Figures/Distal internal rotation 20 fracture/External rotation-scapular/─┌╨220╢╚╜╙╣╟-═Γ╨2-40╢╚.png]

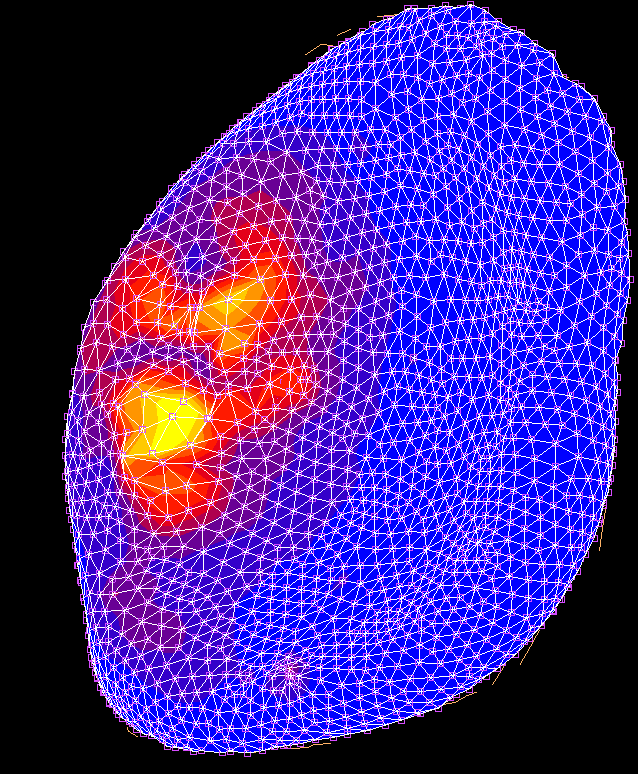

Supplement: Supplementary file 8 — Additional file 8. [file 12891_2022_5479_MOESM8_ESM.zip › Figures/Distal internal rotation 20 fracture/External rotation-scapular/─┌╨220╢╚╜╙╣╟-═Γ╨2-50╢╚.png]

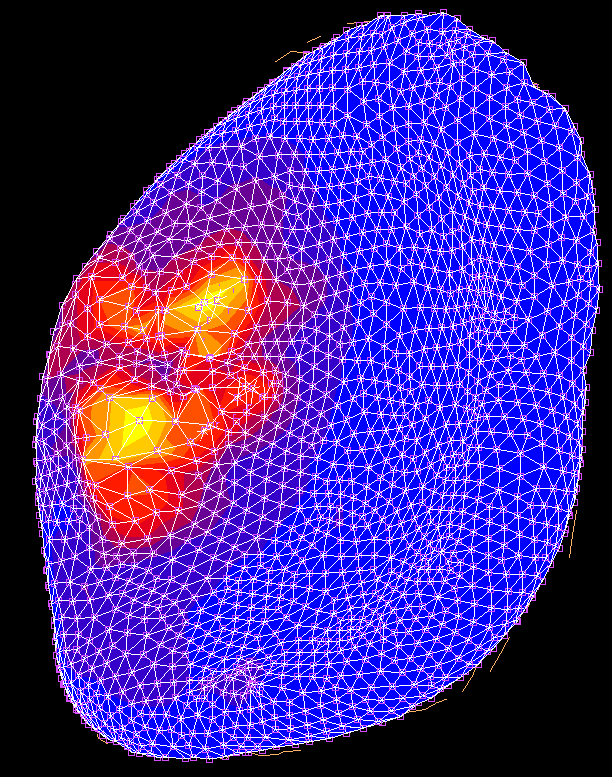

Supplement: Supplementary file 8 — Additional file 8. [file 12891_2022_5479_MOESM8_ESM.zip › Figures/Distal internal rotation 20 fracture/External rotation-scapular/─┌╨220╢╚╜╙╣╟-═Γ╨2-60╢╚.png]

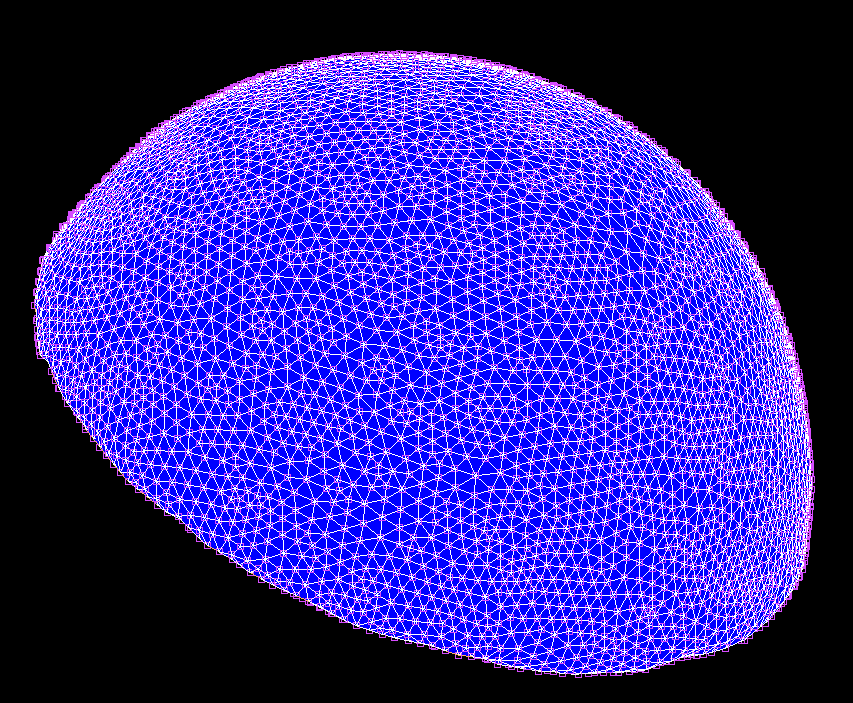

Supplement: Supplementary file 8 — Additional file 8. [file 12891_2022_5479_MOESM8_ESM.zip › Figures/Distal internal rotation 20 fracture/Internal rotation-head/─┌╨220╢╚╜╙╣╟-─┌╨20╢╚.png]

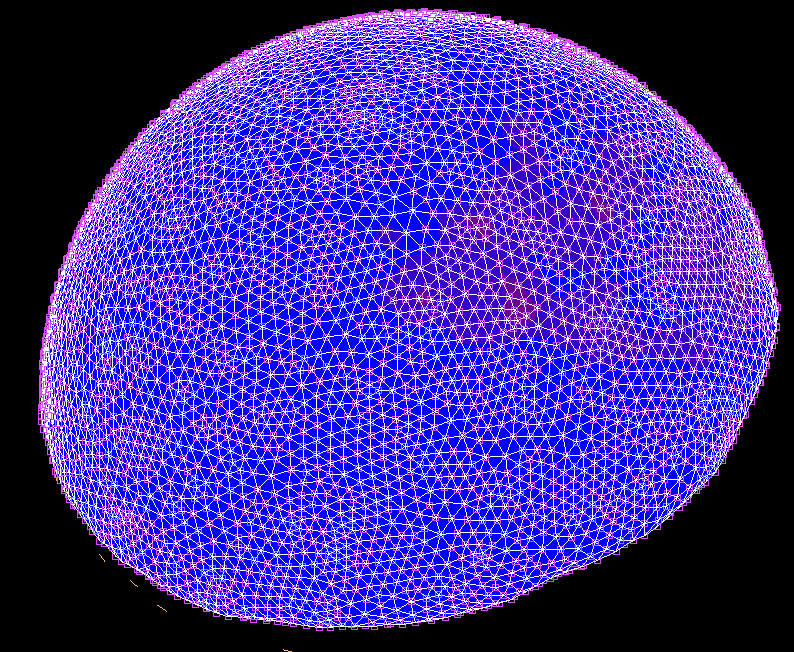

Supplement: Supplementary file 8 — Additional file 8. [file 12891_2022_5479_MOESM8_ESM.zip › Figures/Distal internal rotation 20 fracture/Internal rotation-head/─┌╨220╢╚╜╙╣╟-─┌╨210╢╚.png]

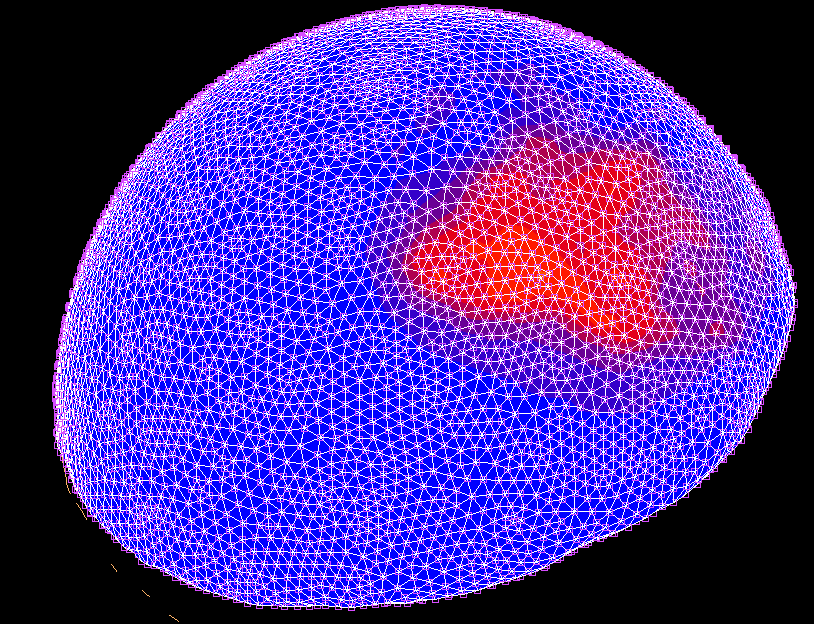

Supplement: Supplementary file 8 — Additional file 8. [file 12891_2022_5479_MOESM8_ESM.zip › Figures/Distal internal rotation 20 fracture/Internal rotation-head/─┌╨220╢╚╜╙╣╟-─┌╨220╢╚.png]

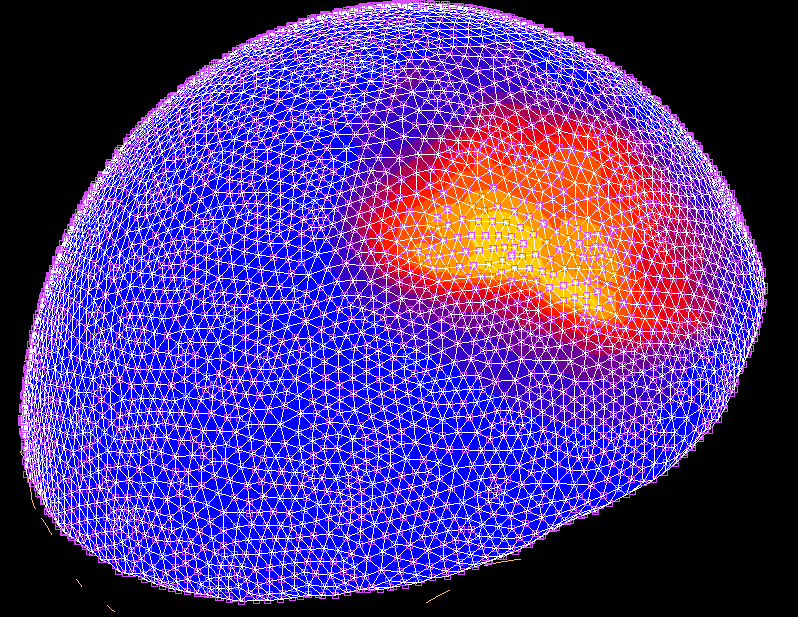

Supplement: Supplementary file 8 — Additional file 8. [file 12891_2022_5479_MOESM8_ESM.zip › Figures/Distal internal rotation 20 fracture/Internal rotation-head/─┌╨220╢╚╜╙╣╟-─┌╨230╢╚.png]

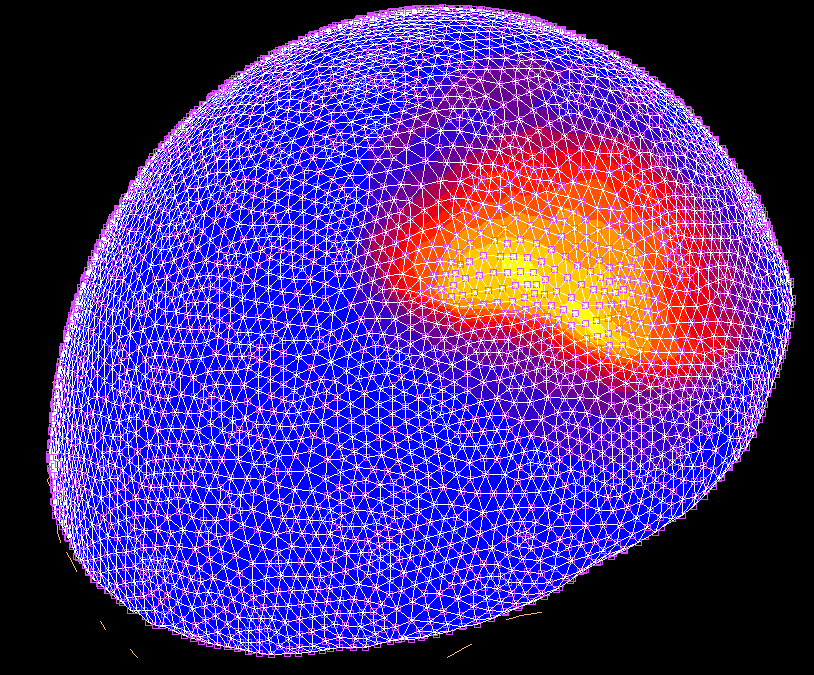

Supplement: Supplementary file 8 — Additional file 8. [file 12891_2022_5479_MOESM8_ESM.zip › Figures/Distal internal rotation 20 fracture/Internal rotation-head/─┌╨220╢╚╜╙╣╟-─┌╨240╢╚.png]

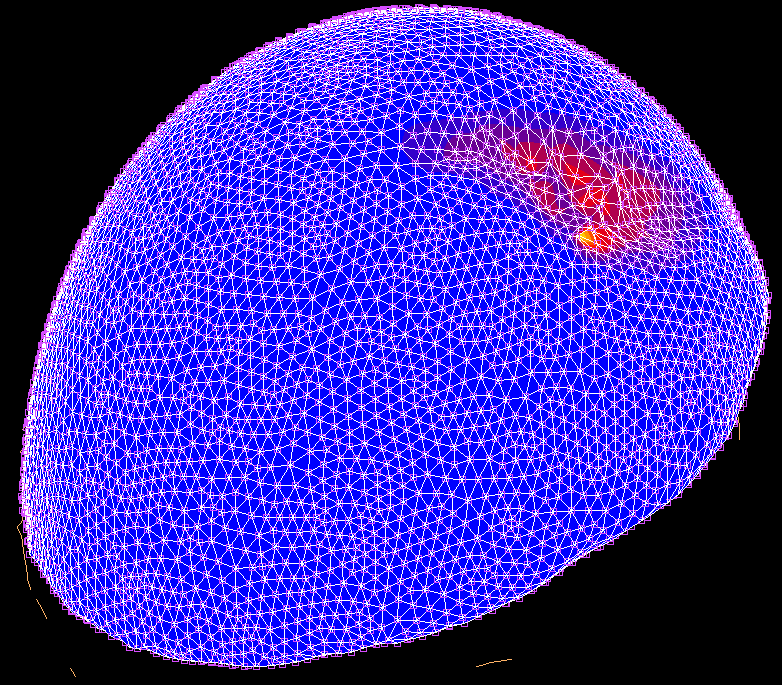

Supplement: Supplementary file 8 — Additional file 8. [file 12891_2022_5479_MOESM8_ESM.zip › Figures/Distal internal rotation 20 fracture/Internal rotation-head/─┌╨220╢╚╜╙╣╟-─┌╨250╢╚.png]

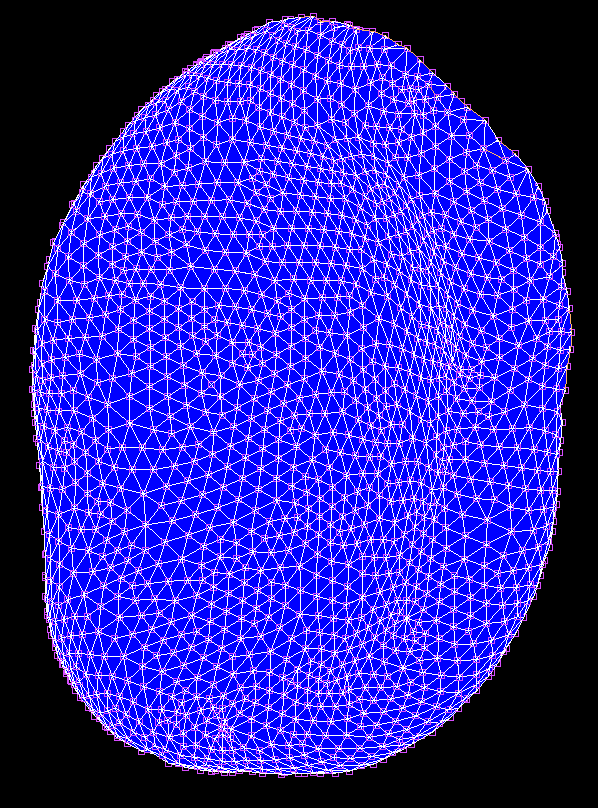

Supplement: Supplementary file 8 — Additional file 8. [file 12891_2022_5479_MOESM8_ESM.zip › Figures/Distal internal rotation 20 fracture/Internal rotation-scapular/─┌╨220╢╚╜╙╣╟-─┌╨20╢╚.png]

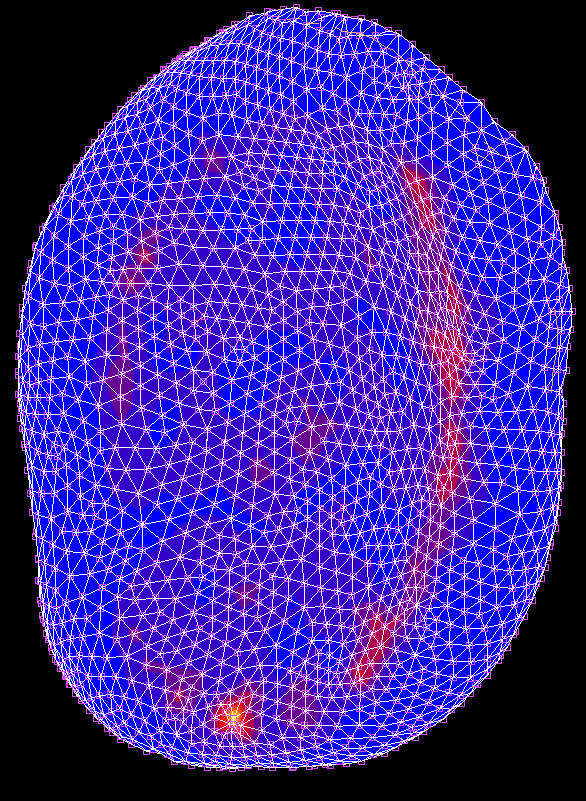

Supplement: Supplementary file 8 — Additional file 8. [file 12891_2022_5479_MOESM8_ESM.zip › Figures/Distal internal rotation 20 fracture/Internal rotation-scapular/─┌╨220╢╚╜╙╣╟-─┌╨210╢╚.png]

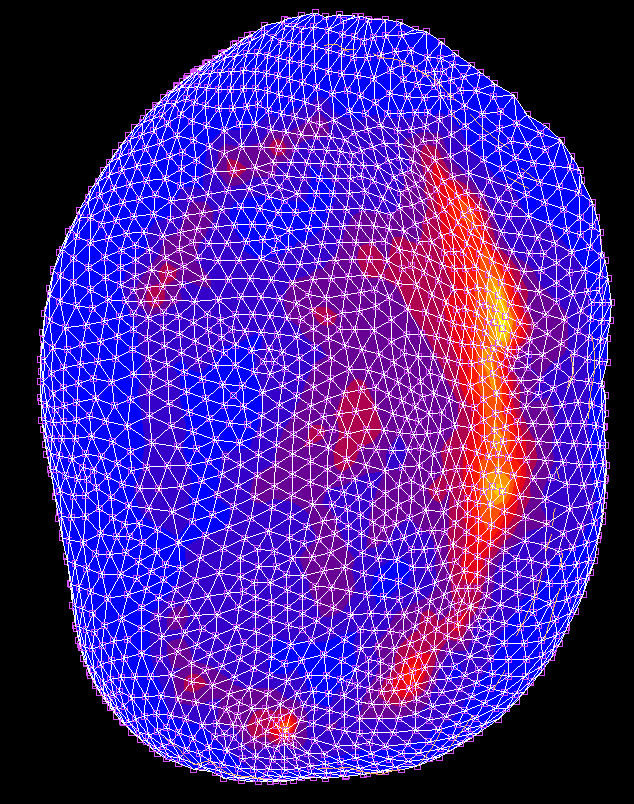

Supplement: Supplementary file 8 — Additional file 8. [file 12891_2022_5479_MOESM8_ESM.zip › Figures/Distal internal rotation 20 fracture/Internal rotation-scapular/─┌╨220╢╚╜╙╣╟-─┌╨220╢╚.png]

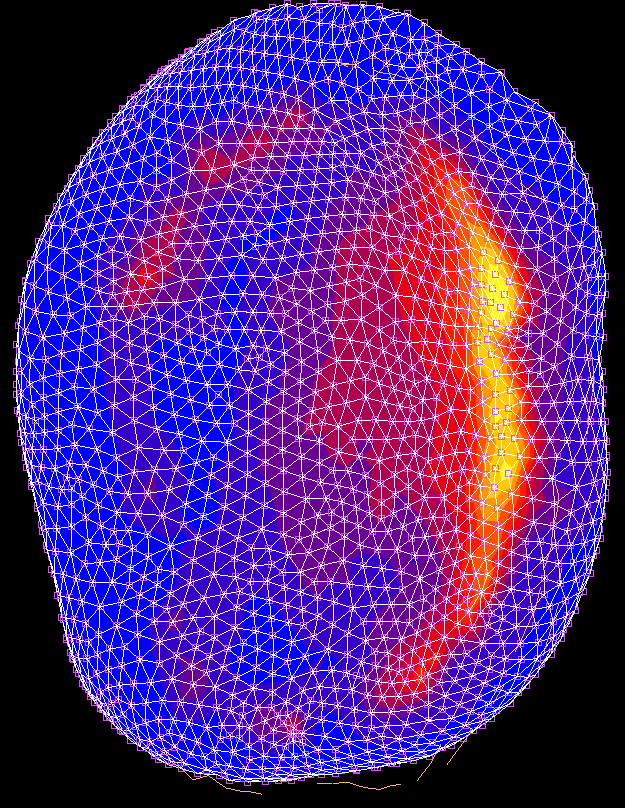

Supplement: Supplementary file 8 — Additional file 8. [file 12891_2022_5479_MOESM8_ESM.zip › Figures/Distal internal rotation 20 fracture/Internal rotation-scapular/─┌╨220╢╚╜╙╣╟-─┌╨230╢╚.png]

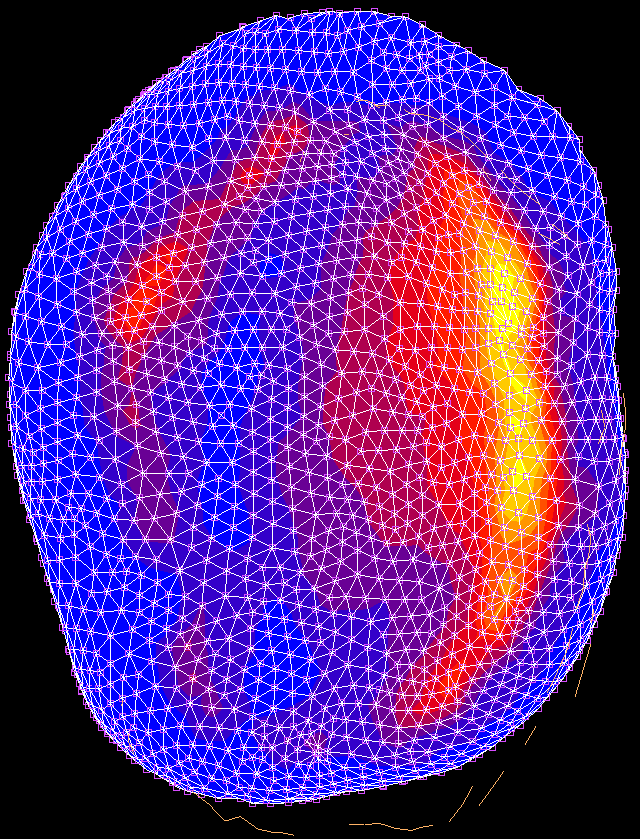

Supplement: Supplementary file 8 — Additional file 8. [file 12891_2022_5479_MOESM8_ESM.zip › Figures/Distal internal rotation 20 fracture/Internal rotation-scapular/─┌╨220╢╚╜╙╣╟-─┌╨240╢╚.png]

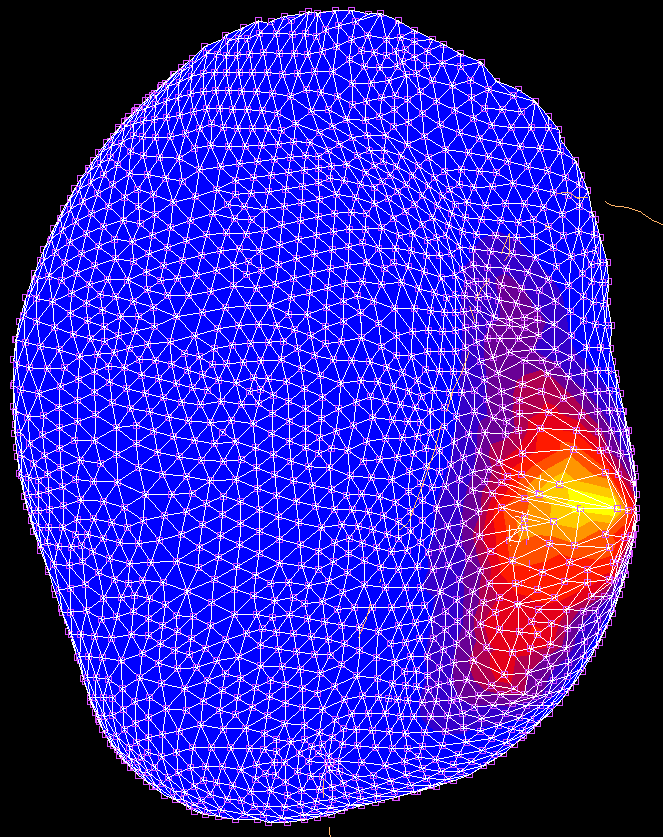

Supplement: Supplementary file 8 — Additional file 8. [file 12891_2022_5479_MOESM8_ESM.zip › Figures/Distal internal rotation 20 fracture/Internal rotation-scapular/─┌╨220╢╚╜╙╣╟-─┌╨250╢╚.png]

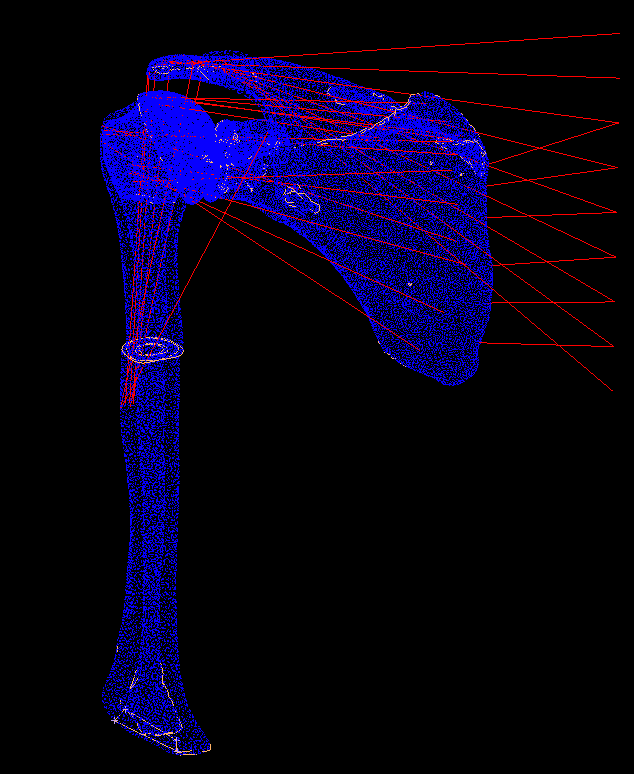

Supplement: Supplementary file 8 — Additional file 8. [file 12891_2022_5479_MOESM8_ESM.zip › Figures/Distal internal rotation 20 fracture/Model.png]

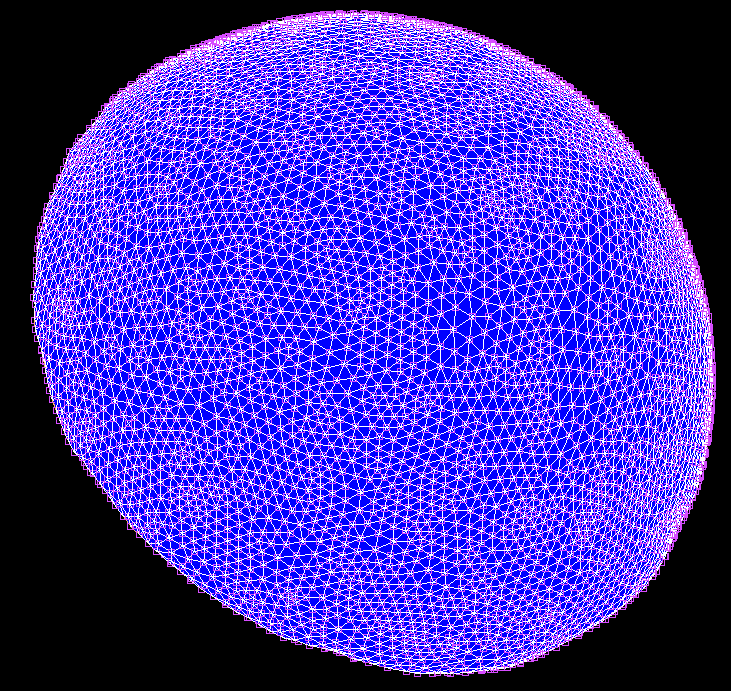

Supplement: Supplementary file 8 — Additional file 8. [file 12891_2022_5479_MOESM8_ESM.zip › Figures/Normal/External rotation 60-head/╒2│ú═Γ╨2í¬0╢╚-═╖.png]

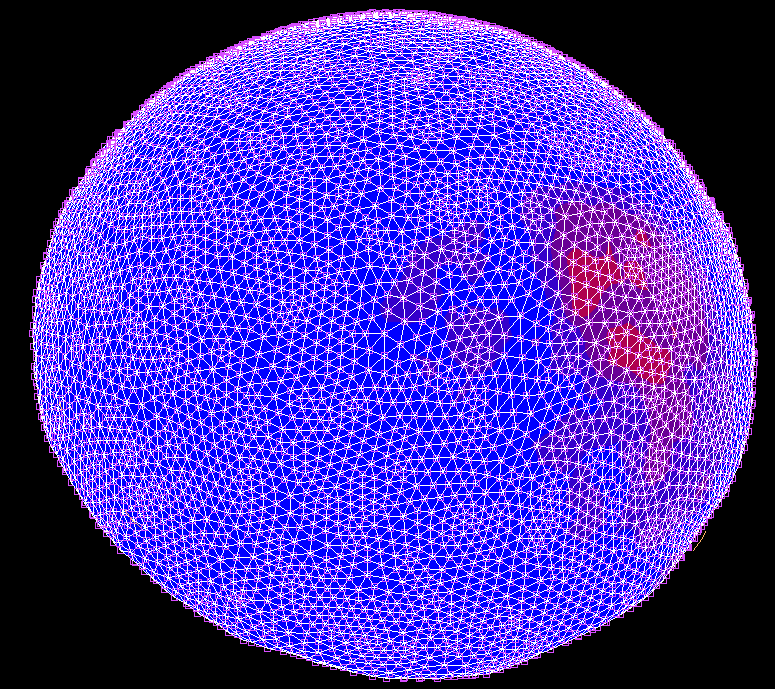

Supplement: Supplementary file 8 — Additional file 8. [file 12891_2022_5479_MOESM8_ESM.zip › Figures/Normal/External rotation 60-head/╒2│ú═Γ╨2í¬10╢╚-═╖.png]

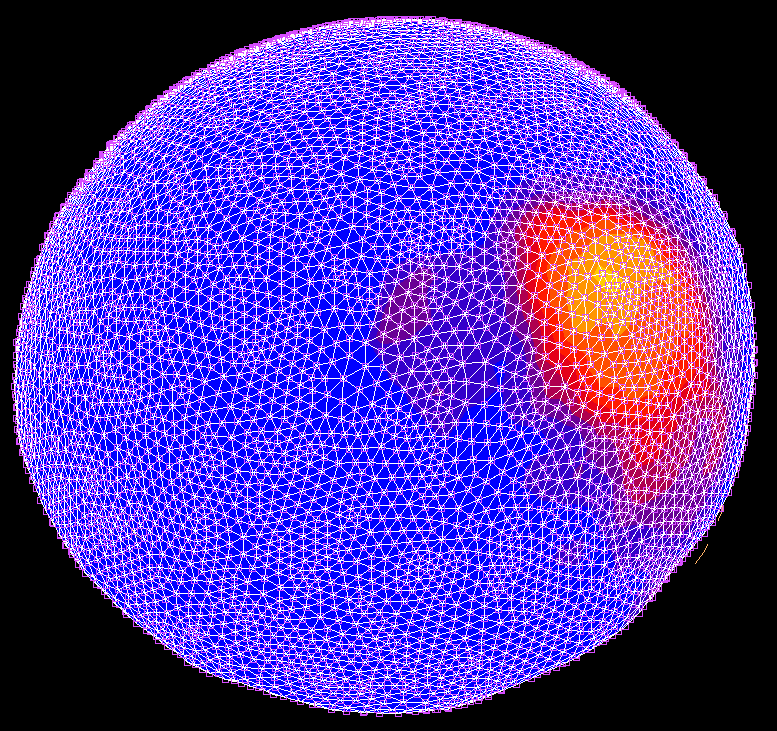

Supplement: Supplementary file 8 — Additional file 8. [file 12891_2022_5479_MOESM8_ESM.zip › Figures/Normal/External rotation 60-head/╒2│ú═Γ╨2í¬20╢╚-═╖.png]

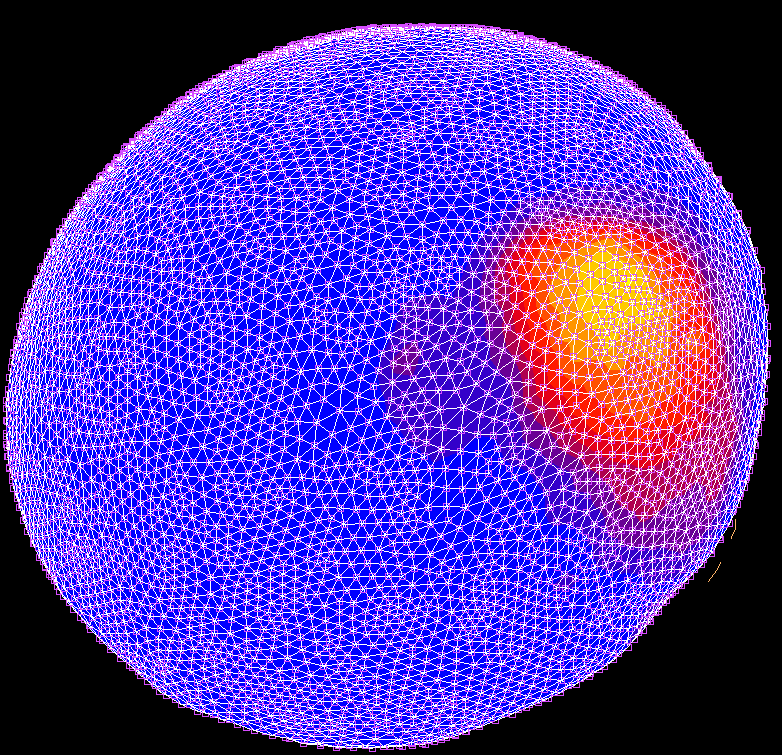

Supplement: Supplementary file 8 — Additional file 8. [file 12891_2022_5479_MOESM8_ESM.zip › Figures/Normal/External rotation 60-head/╒2│ú═Γ╨2í¬30╢╚-═╖.png]

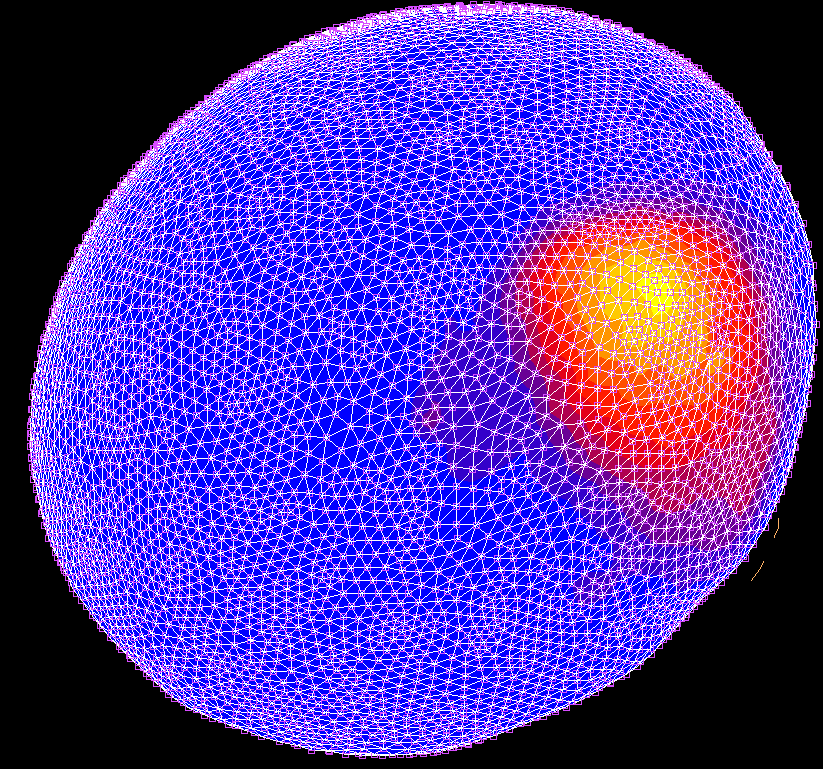

Supplement: Supplementary file 8 — Additional file 8. [file 12891_2022_5479_MOESM8_ESM.zip › Figures/Normal/External rotation 60-head/╒2│ú═Γ╨2í¬40╢╚-═╖.png]

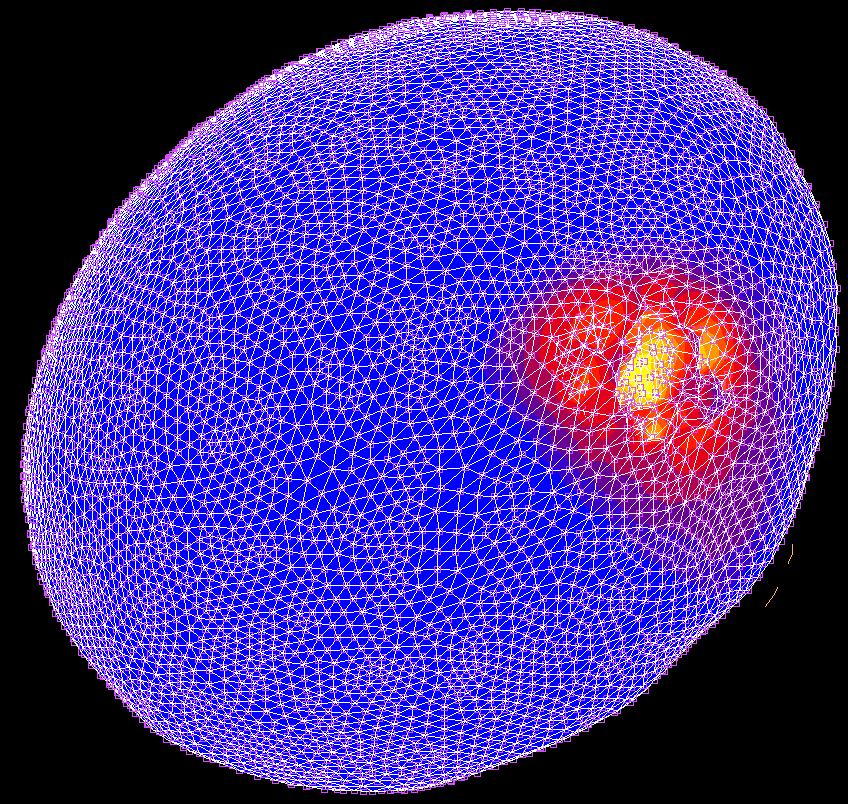

Supplement: Supplementary file 8 — Additional file 8. [file 12891_2022_5479_MOESM8_ESM.zip › Figures/Normal/External rotation 60-head/╒2│ú═Γ╨2í¬50╢╚-═╖.png]

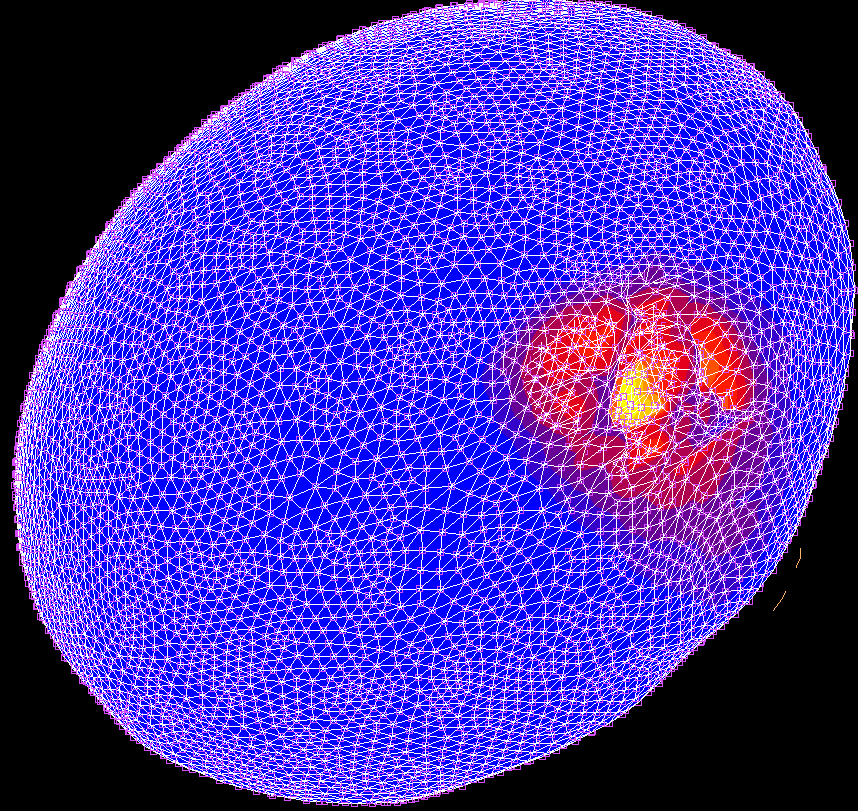

Supplement: Supplementary file 8 — Additional file 8. [file 12891_2022_5479_MOESM8_ESM.zip › Figures/Normal/External rotation 60-head/╒2│ú═Γ╨2í¬60╢╚-═╖.png]

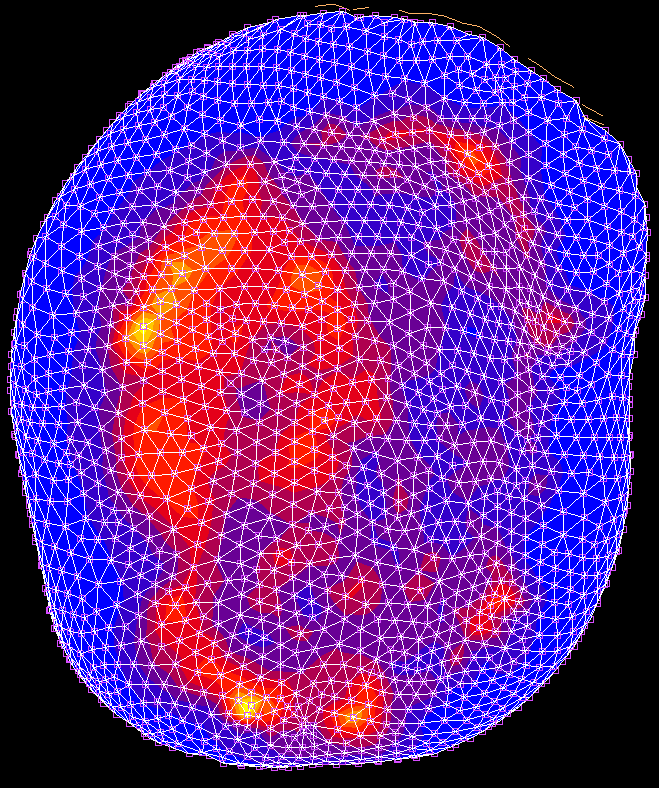

Supplement: Supplementary file 8 — Additional file 8. [file 12891_2022_5479_MOESM8_ESM.zip › Figures/Normal/External rotation 60-scapular/╒2│ú═Γ╨2_10╢╚.png]

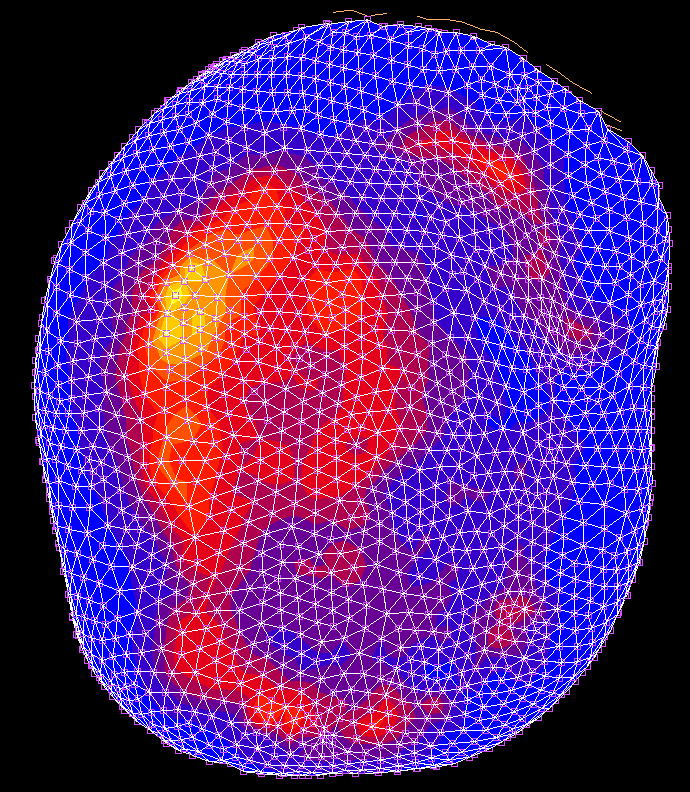

Supplement: Supplementary file 8 — Additional file 8. [file 12891_2022_5479_MOESM8_ESM.zip › Figures/Normal/External rotation 60-scapular/╒2│ú═Γ╨2_15╢╚.png]

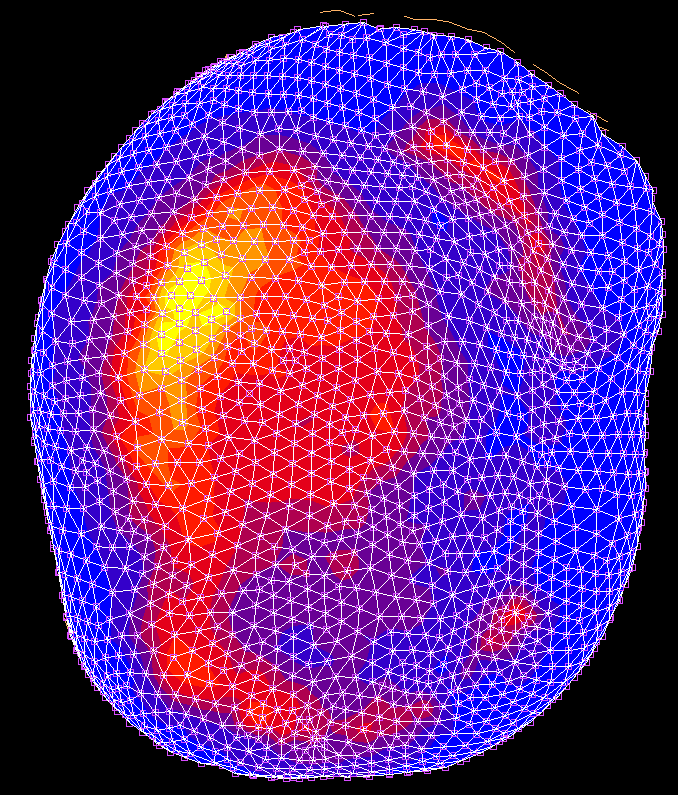

Supplement: Supplementary file 8 — Additional file 8. [file 12891_2022_5479_MOESM8_ESM.zip › Figures/Normal/External rotation 60-scapular/╒2│ú═Γ╨2_20╢╚.png]

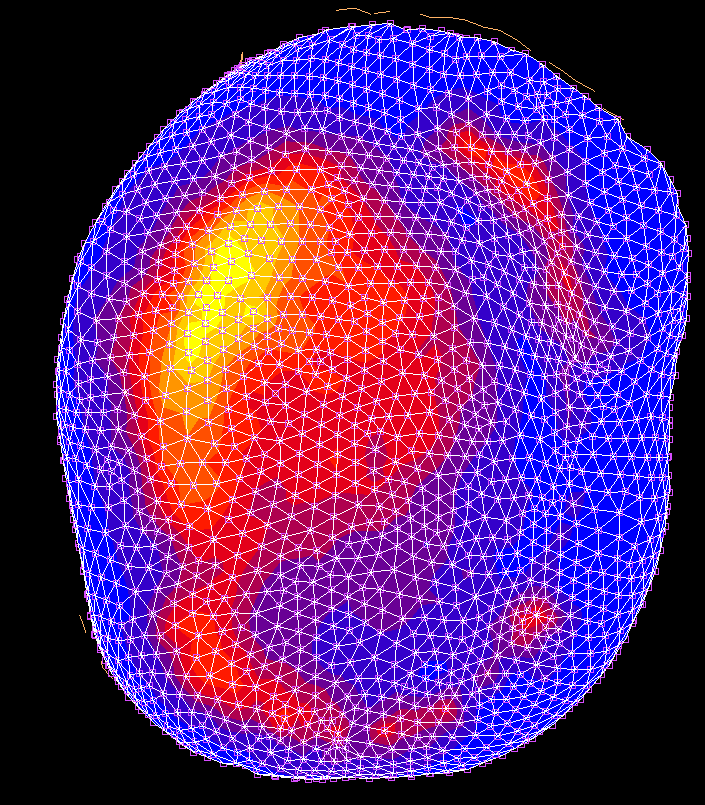

Supplement: Supplementary file 8 — Additional file 8. [file 12891_2022_5479_MOESM8_ESM.zip › Figures/Normal/External rotation 60-scapular/╒2│ú═Γ╨2_25╢╚.png]

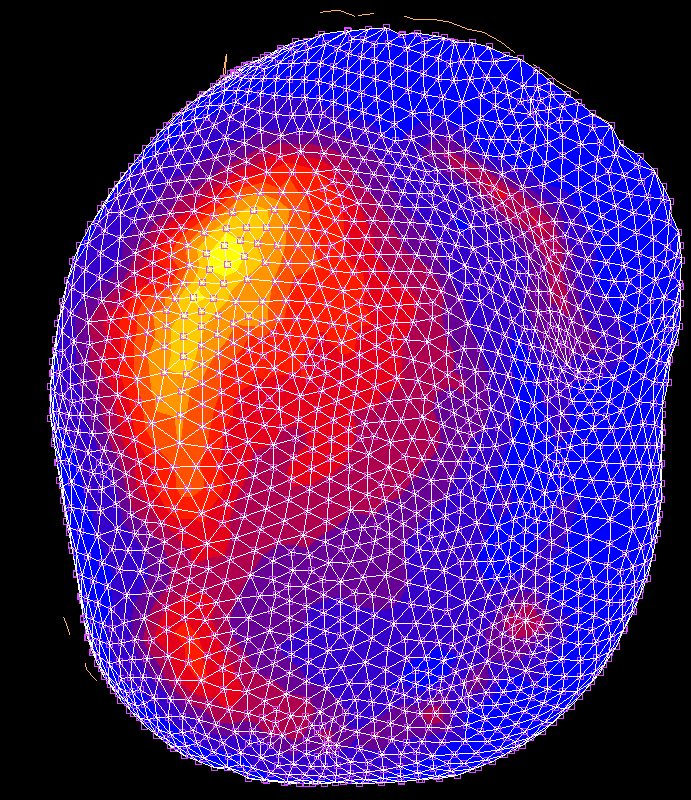

Supplement: Supplementary file 8 — Additional file 8. [file 12891_2022_5479_MOESM8_ESM.zip › Figures/Normal/External rotation 60-scapular/╒2│ú═Γ╨2_30╢╚.png]

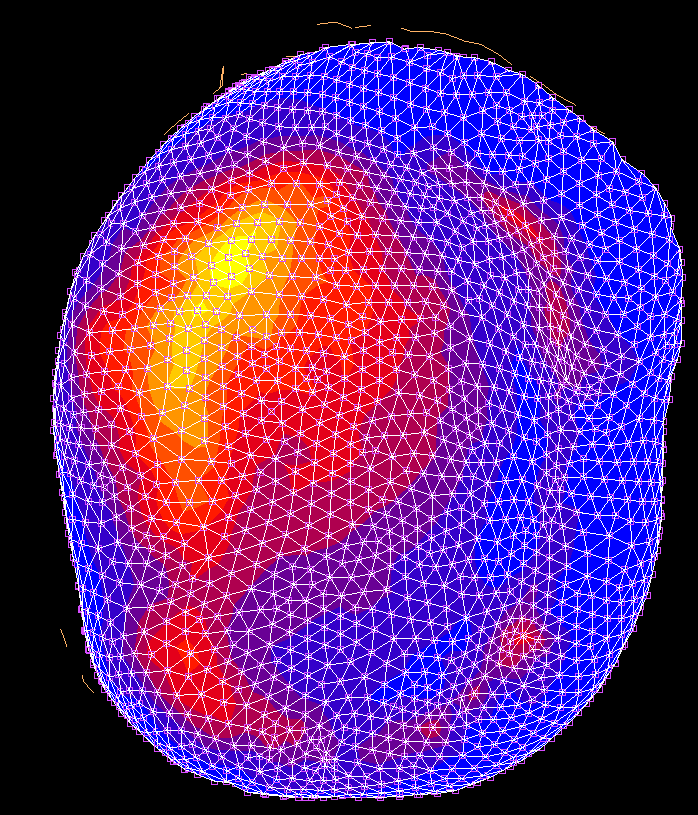

Supplement: Supplementary file 8 — Additional file 8. [file 12891_2022_5479_MOESM8_ESM.zip › Figures/Normal/External rotation 60-scapular/╒2│ú═Γ╨2_35╢╚.png]

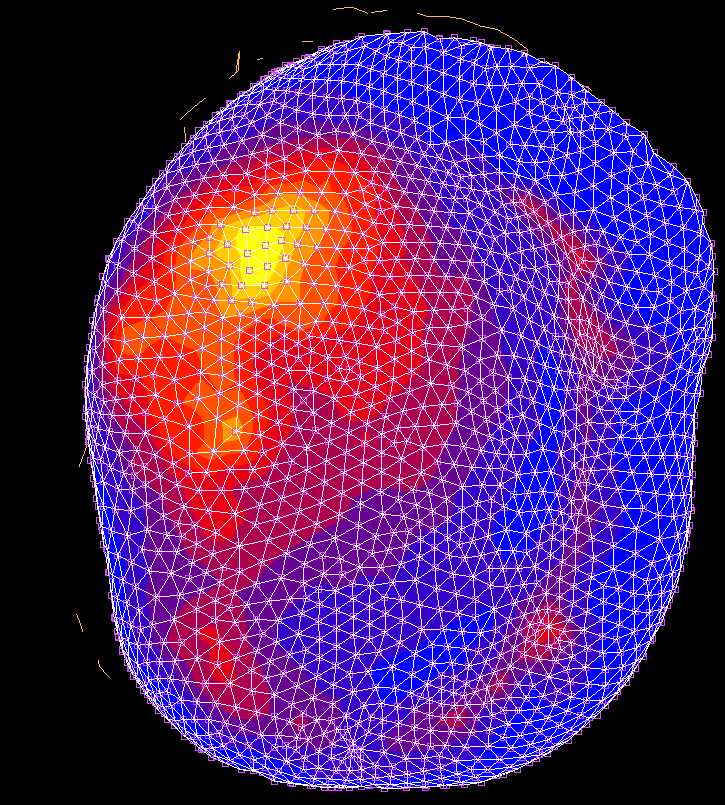

Supplement: Supplementary file 8 — Additional file 8. [file 12891_2022_5479_MOESM8_ESM.zip › Figures/Normal/External rotation 60-scapular/╒2│ú═Γ╨2_40╢╚.png]

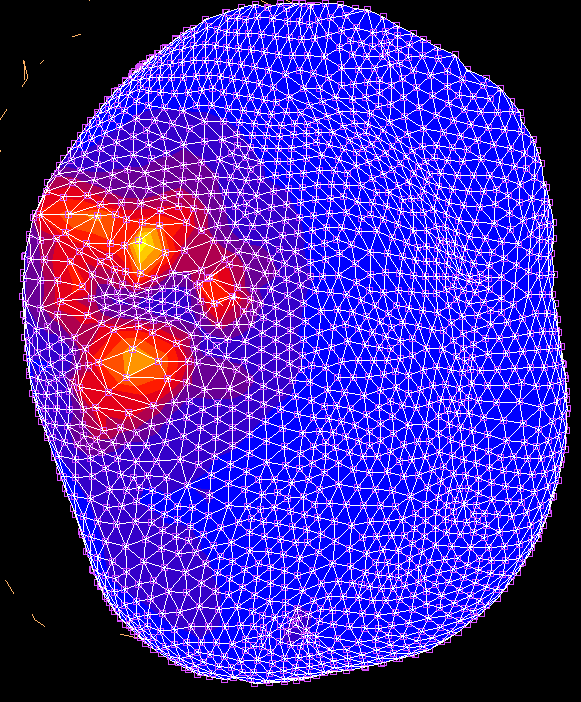

Supplement: Supplementary file 8 — Additional file 8. [file 12891_2022_5479_MOESM8_ESM.zip › Figures/Normal/External rotation 60-scapular/╒2│ú═Γ╨2_50╢╚.png]

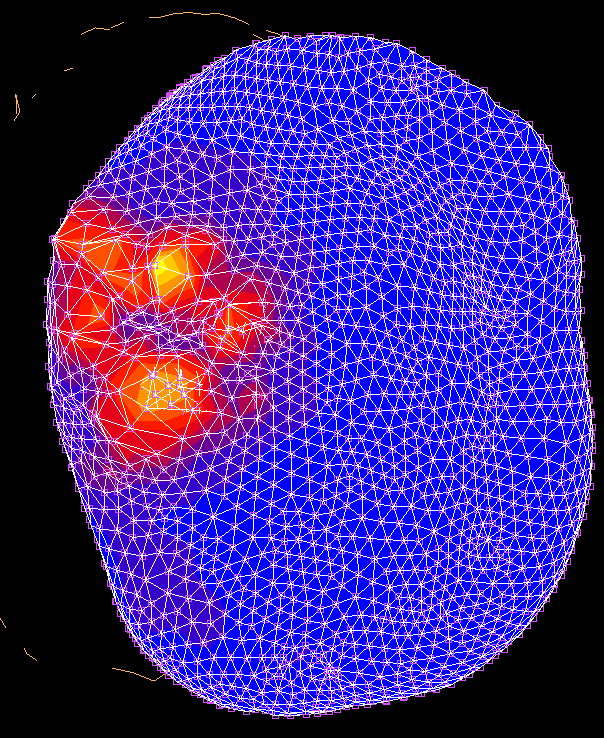

Supplement: Supplementary file 8 — Additional file 8. [file 12891_2022_5479_MOESM8_ESM.zip › Figures/Normal/External rotation 60-scapular/╒2│ú═Γ╨2_60╢╚.png]

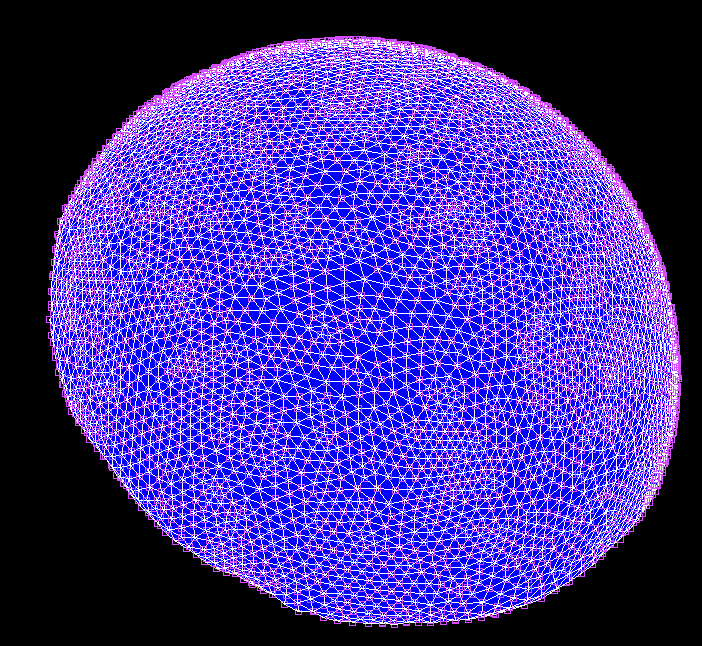

Supplement: Supplementary file 8 — Additional file 8. [file 12891_2022_5479_MOESM8_ESM.zip › Figures/Normal/Internal rotation 60-head/╒2│ú─┌╨2_0╢╚.png]

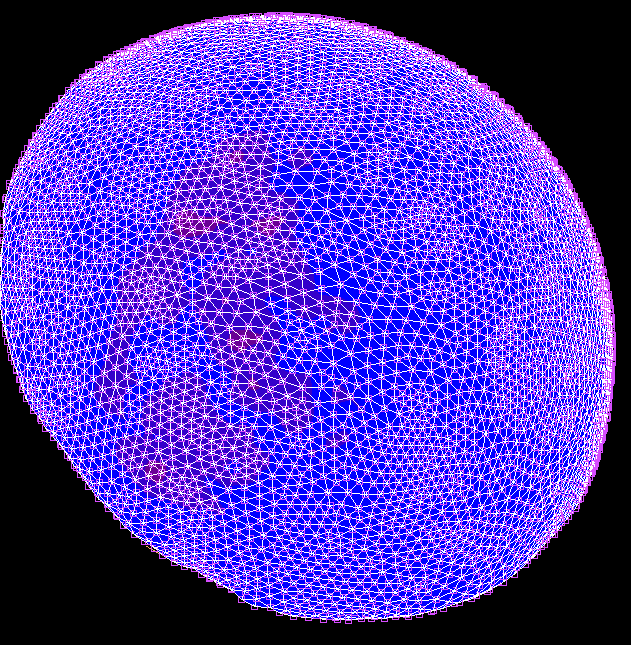

Supplement: Supplementary file 8 — Additional file 8. [file 12891_2022_5479_MOESM8_ESM.zip › Figures/Normal/Internal rotation 60-head/╒2│ú─┌╨2_10╢╚.png]

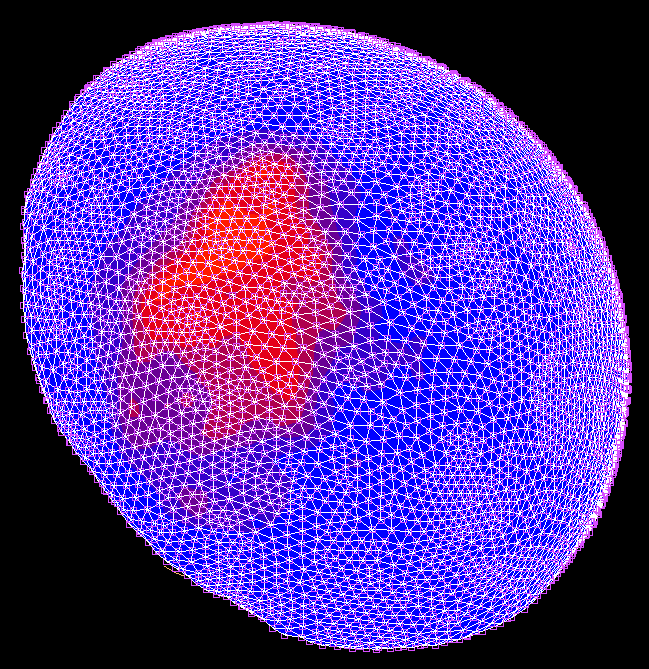

Supplement: Supplementary file 8 — Additional file 8. [file 12891_2022_5479_MOESM8_ESM.zip › Figures/Normal/Internal rotation 60-head/╒2│ú─┌╨2_20╢╚.png]

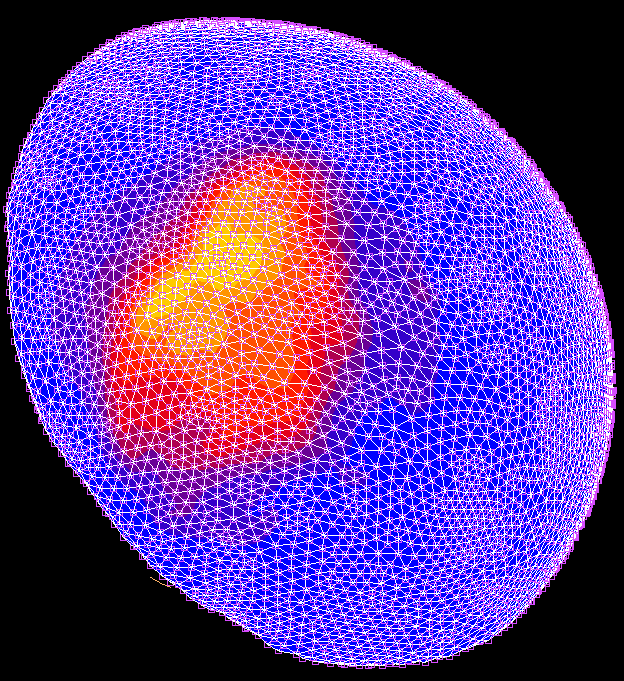

Supplement: Supplementary file 8 — Additional file 8. [file 12891_2022_5479_MOESM8_ESM.zip › Figures/Normal/Internal rotation 60-head/╒2│ú─┌╨2_30╢╚.png]

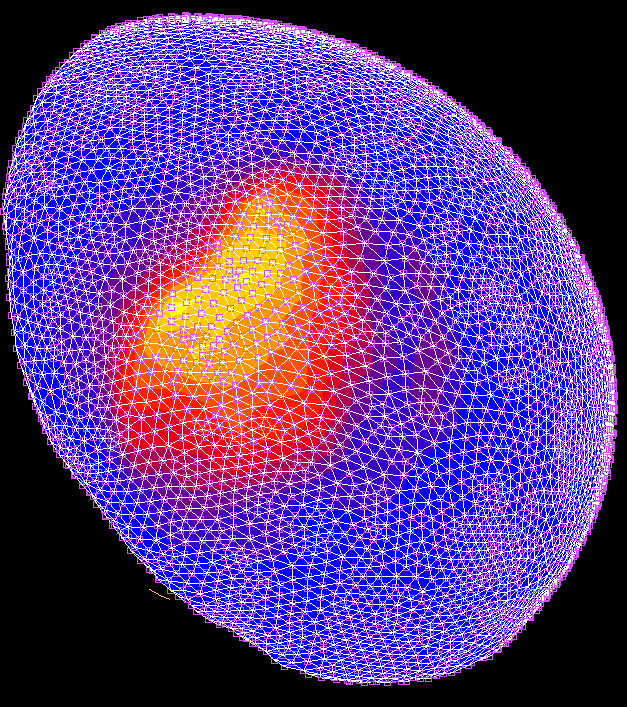

Supplement: Supplementary file 8 — Additional file 8. [file 12891_2022_5479_MOESM8_ESM.zip › Figures/Normal/Internal rotation 60-head/╒2│ú─┌╨2_40╢╚.png]

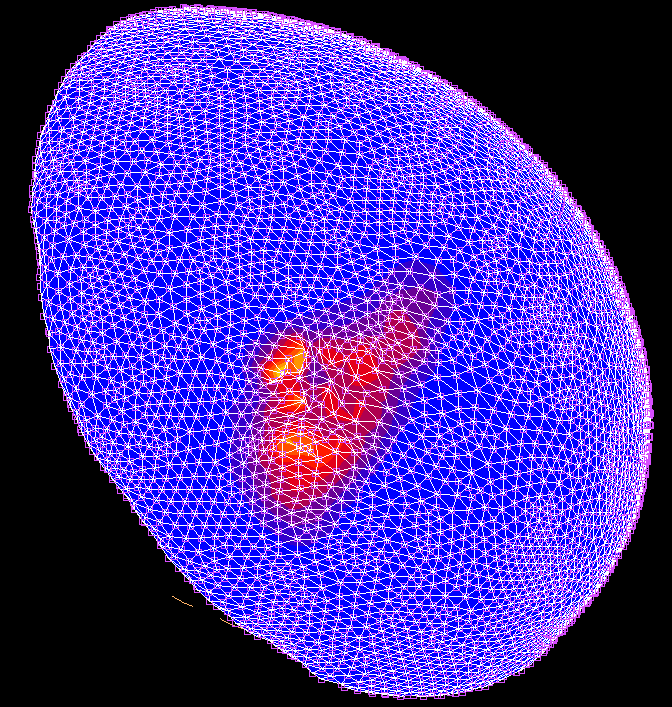

Supplement: Supplementary file 8 — Additional file 8. [file 12891_2022_5479_MOESM8_ESM.zip › Figures/Normal/Internal rotation 60-head/╒2│ú─┌╨2_50╢╚.png]

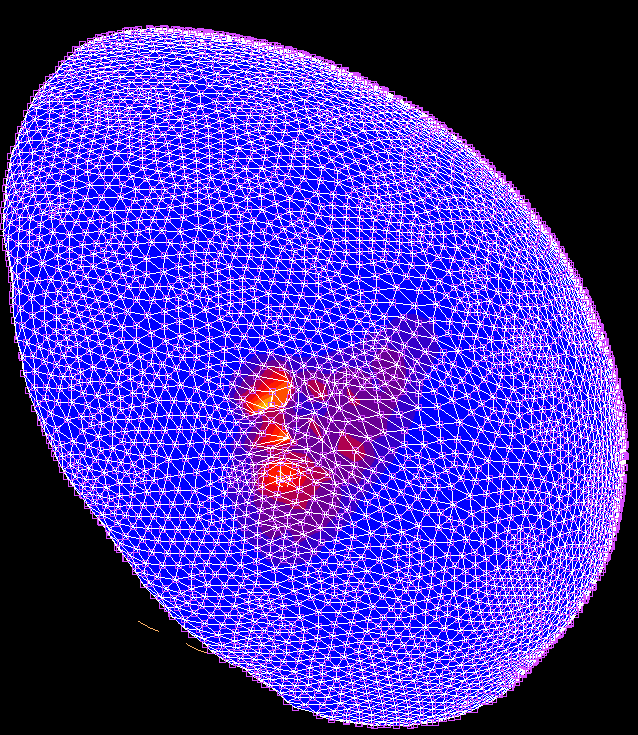

Supplement: Supplementary file 8 — Additional file 8. [file 12891_2022_5479_MOESM8_ESM.zip › Figures/Normal/Internal rotation 60-head/╒2│ú─┌╨2_60╢╚.png]

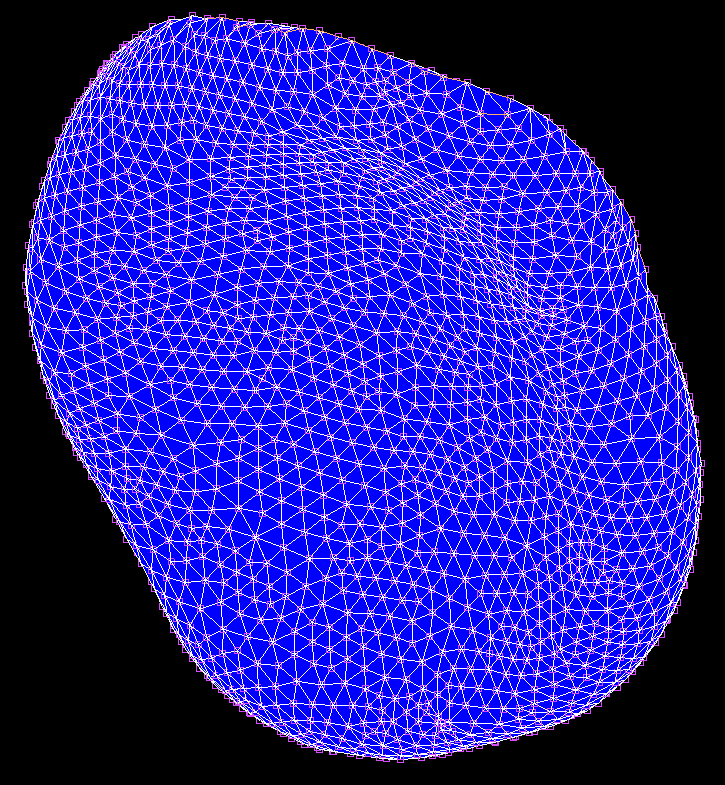

Supplement: Supplementary file 8 — Additional file 8. [file 12891_2022_5479_MOESM8_ESM.zip › Figures/Normal/Internal rotation 60-scapular/╒2│ú─┌╨260╢╚_0╢╚.png]

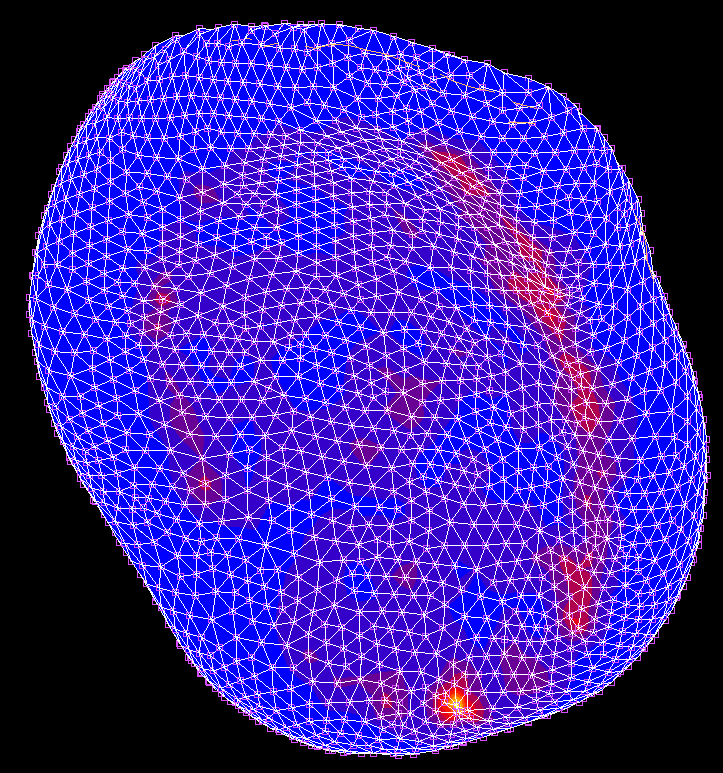

Supplement: Supplementary file 8 — Additional file 8. [file 12891_2022_5479_MOESM8_ESM.zip › Figures/Normal/Internal rotation 60-scapular/╒2│ú─┌╨260╢╚_10╢╚.png]

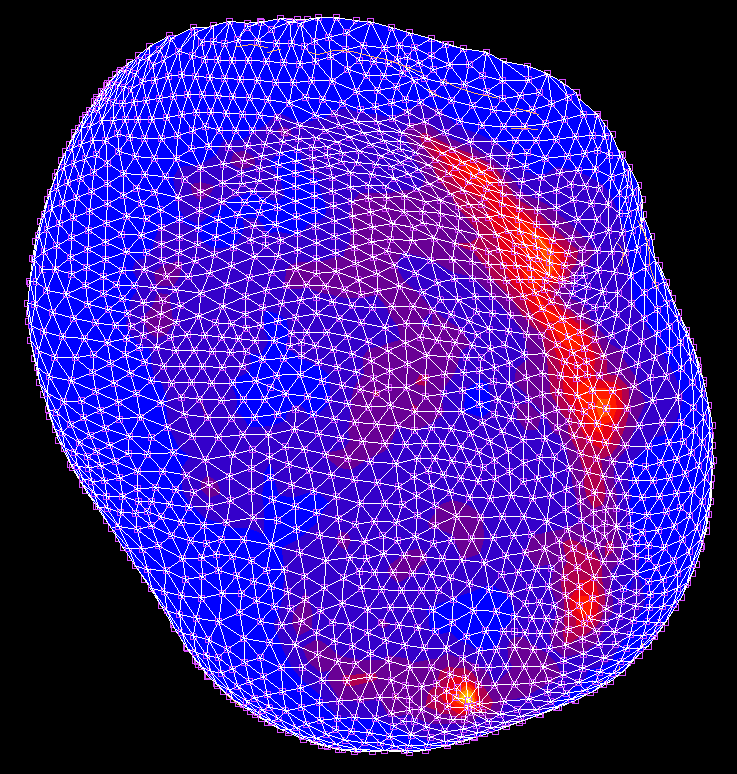

Supplement: Supplementary file 8 — Additional file 8. [file 12891_2022_5479_MOESM8_ESM.zip › Figures/Normal/Internal rotation 60-scapular/╒2│ú─┌╨260╢╚_15╢╚.png]

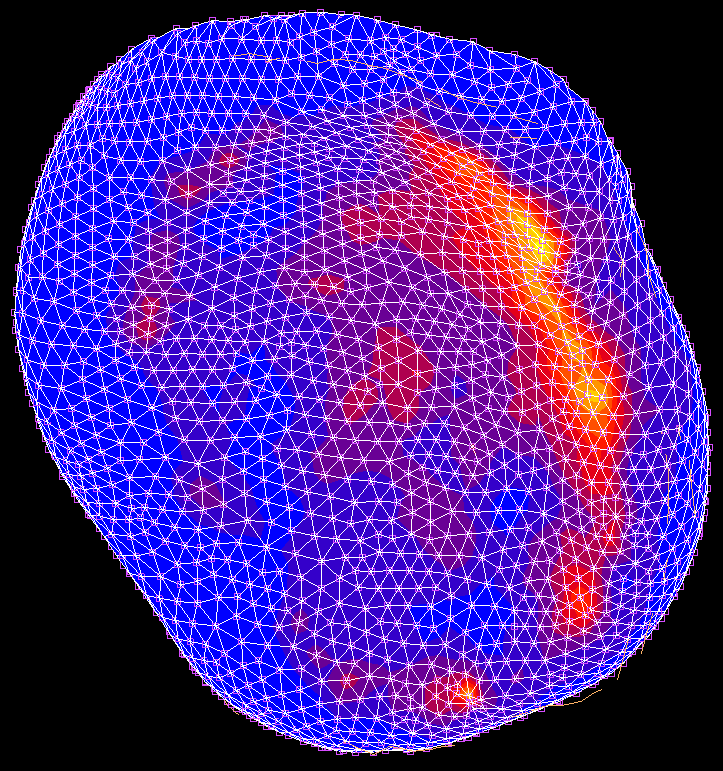

Supplement: Supplementary file 8 — Additional file 8. [file 12891_2022_5479_MOESM8_ESM.zip › Figures/Normal/Internal rotation 60-scapular/╒2│ú─┌╨260╢╚_20╢╚.png]

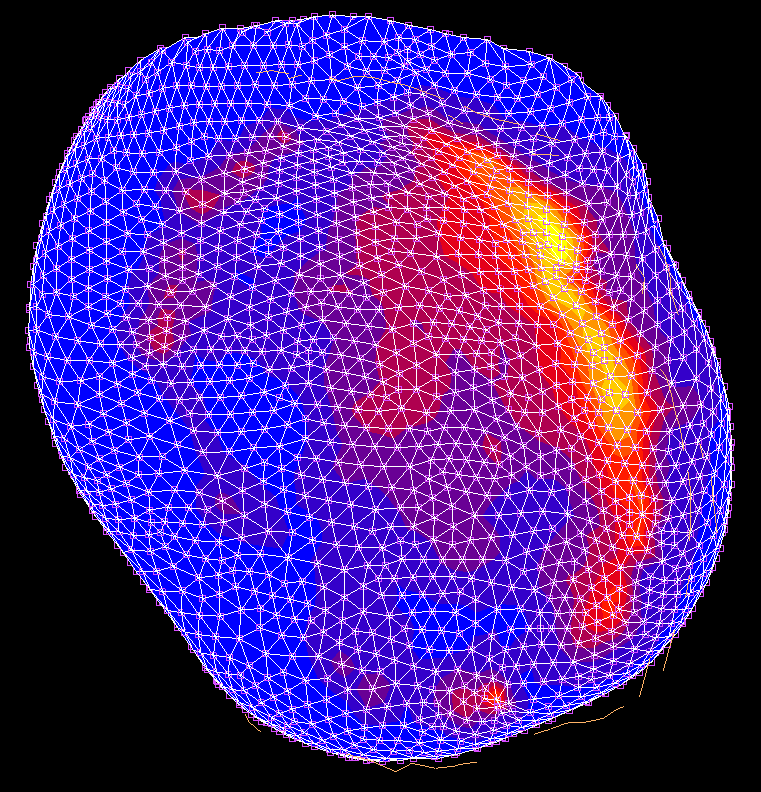

Supplement: Supplementary file 8 — Additional file 8. [file 12891_2022_5479_MOESM8_ESM.zip › Figures/Normal/Internal rotation 60-scapular/╒2│ú─┌╨260╢╚_25╢╚.png]

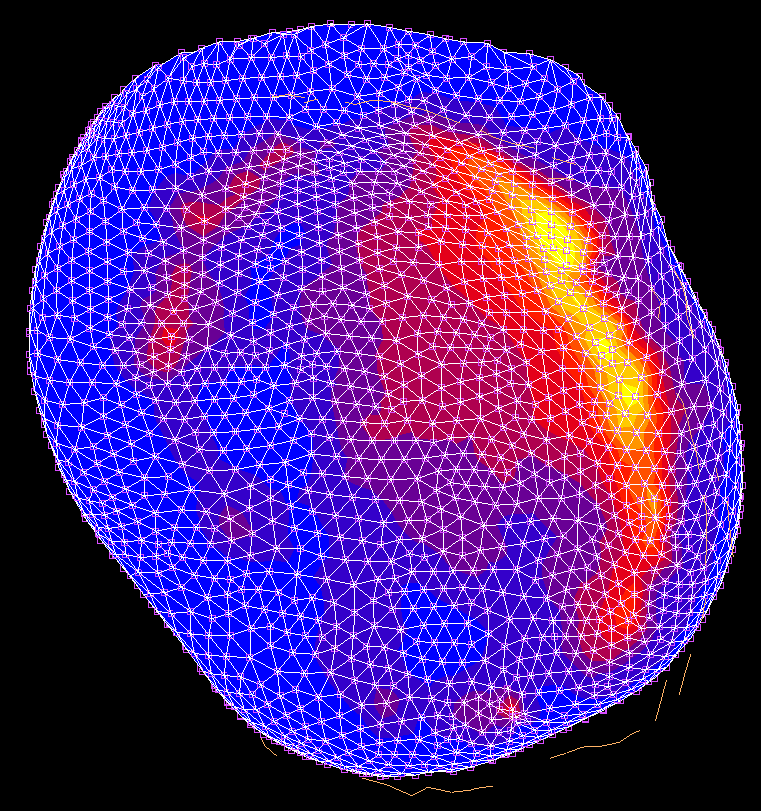

Supplement: Supplementary file 8 — Additional file 8. [file 12891_2022_5479_MOESM8_ESM.zip › Figures/Normal/Internal rotation 60-scapular/╒2│ú─┌╨260╢╚_30╢╚.png]

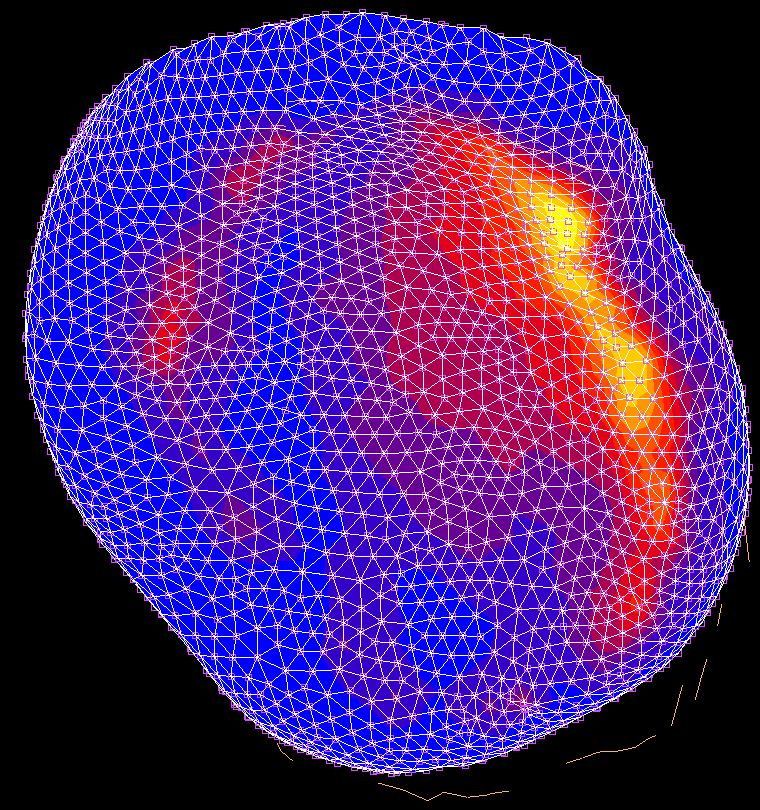

Supplement: Supplementary file 8 — Additional file 8. [file 12891_2022_5479_MOESM8_ESM.zip › Figures/Normal/Internal rotation 60-scapular/╒2│ú─┌╨260╢╚_35╢╚.png]

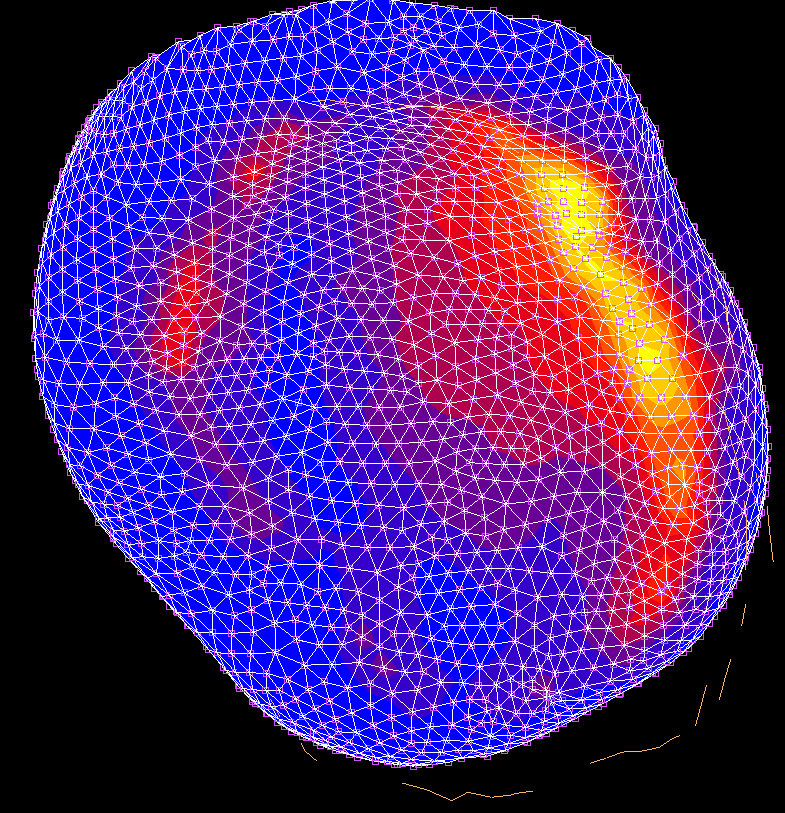

Supplement: Supplementary file 8 — Additional file 8. [file 12891_2022_5479_MOESM8_ESM.zip › Figures/Normal/Internal rotation 60-scapular/╒2│ú─┌╨260╢╚_40╢╚.png]

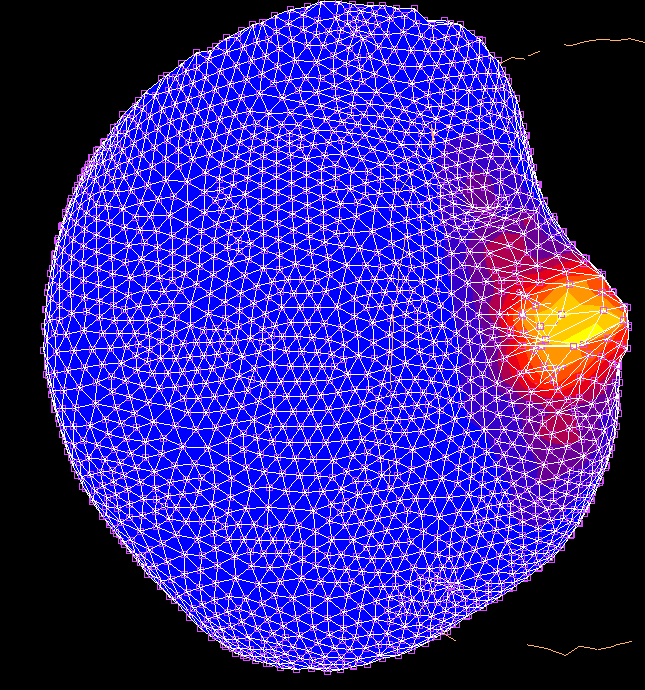

Supplement: Supplementary file 8 — Additional file 8. [file 12891_2022_5479_MOESM8_ESM.zip › Figures/Normal/Internal rotation 60-scapular/╒2│ú─┌╨260╢╚_50╢╚.png]

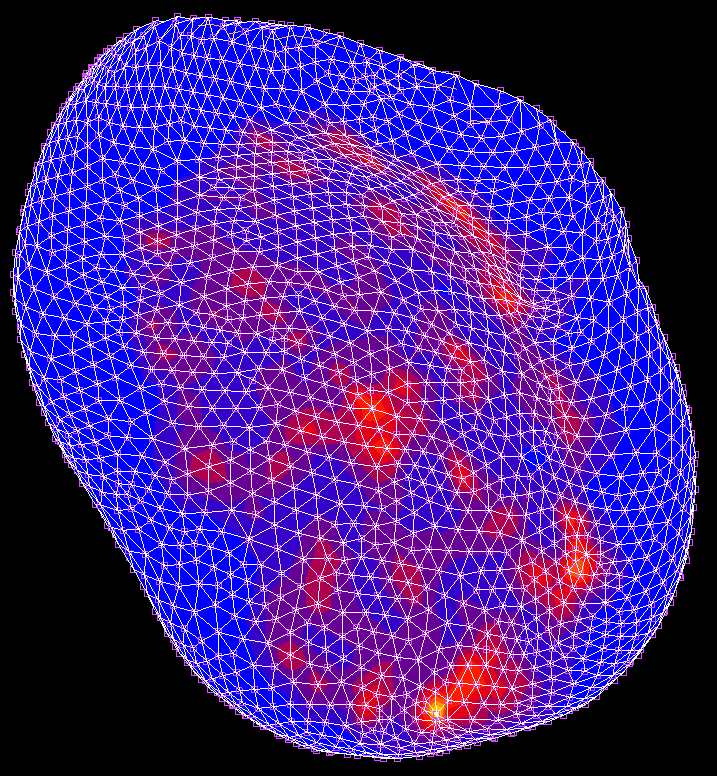

Supplement: Supplementary file 8 — Additional file 8. [file 12891_2022_5479_MOESM8_ESM.zip › Figures/Normal/Internal rotation 60-scapular/╒2│ú─┌╨260╢╚_5╢╚.png]

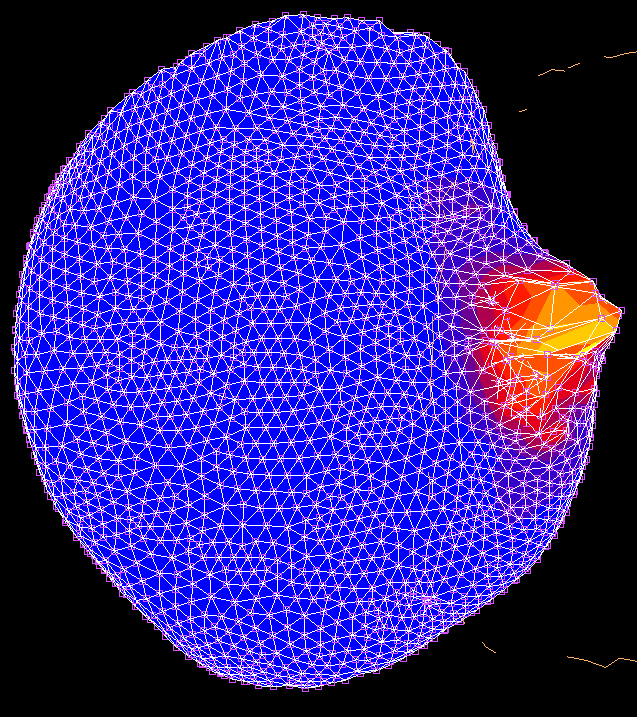

Supplement: Supplementary file 8 — Additional file 8. [file 12891_2022_5479_MOESM8_ESM.zip › Figures/Normal/Internal rotation 60-scapular/╒2│ú─┌╨260╢╚_60╢╚.png]

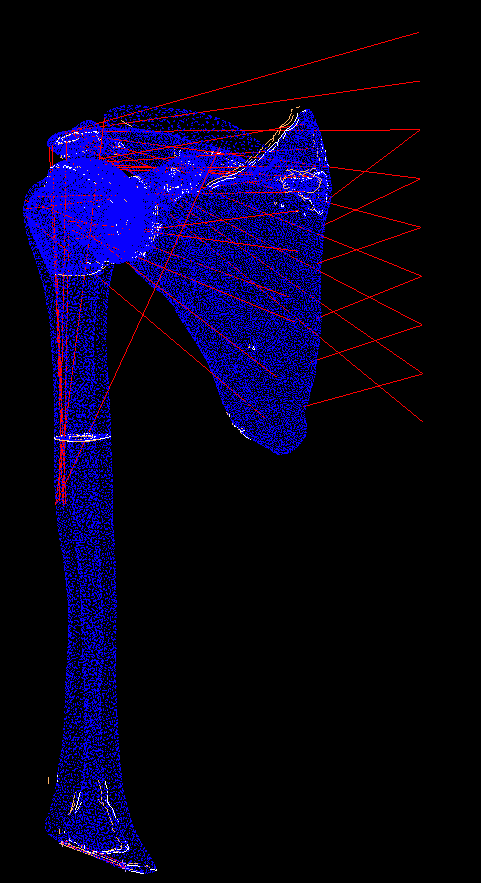

Supplement: Supplementary file 8 — Additional file 8. [file 12891_2022_5479_MOESM8_ESM.zip › Figures/Normal/Model.png]

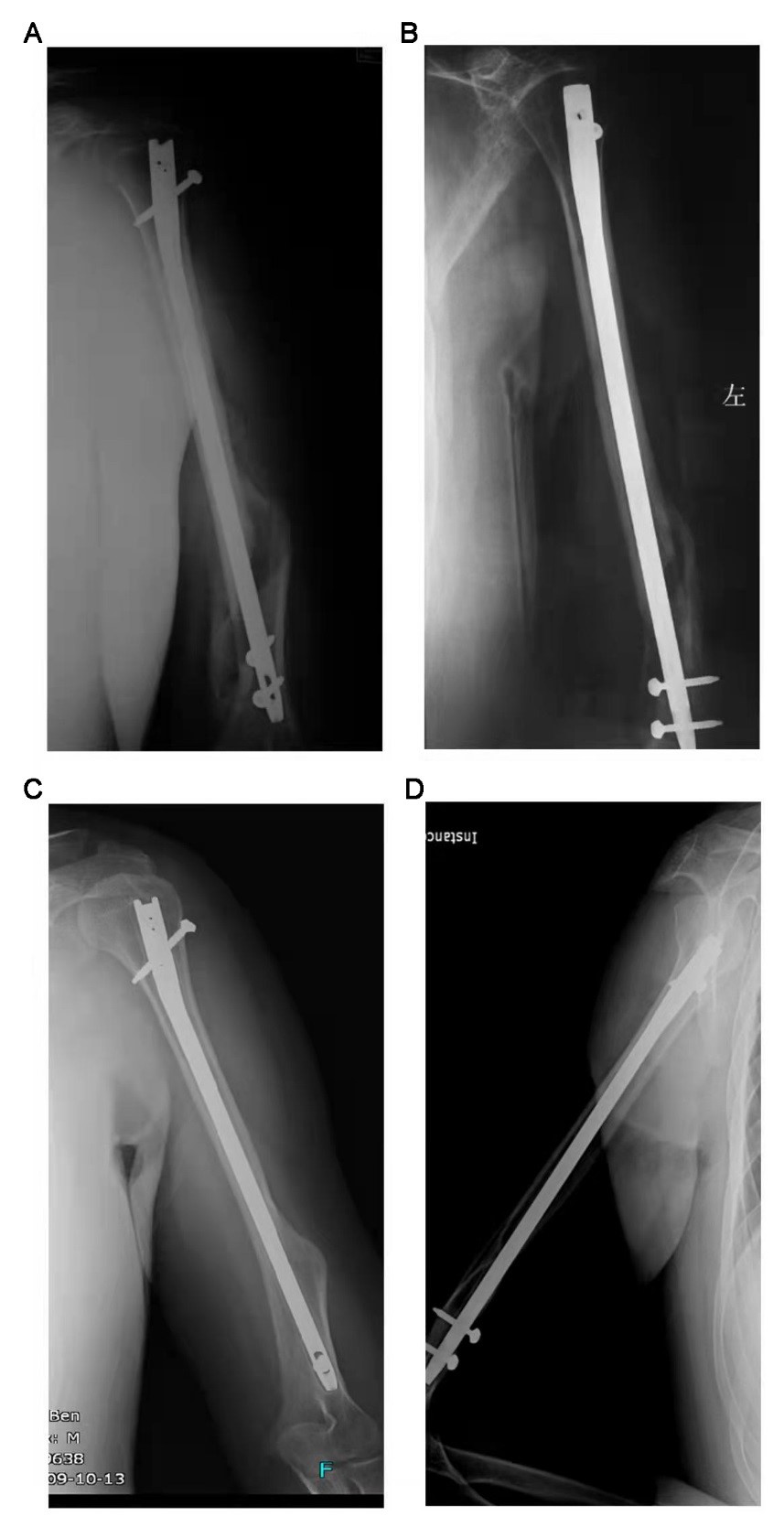

Supplement: Supplementary file 8 — Additional file 8. [file 12891_2022_5479_MOESM8_ESM.zip › Figures/Supplementary figure 1.jpg]
