# Supplementary material for: A finite element model of the shoulder: application to the changes of biomechanical environment induced by postoperative malrotation of humeral shaft fracture
Source: BMC Musculoskelet Disord. 2022 Jun 2;23:525. doi: 10.1186/s12891-022-05479-3 (PMC9161483; doi:10.1186/s12891-022-05479-3)
Supplement: Supplementary file 3 — Additional file 3. [file 12891_2022_5479_MOESM3_ESM.zip › Abacus/╝╞╦π─┌╚▌.pdf]

No.	文件名(.mud/.t16)	初始状态	工况		软骨应力	旋转中心
1	normal_in_ro_60	完全正常接骨	内旋60			
2	normal_ex_ro_60		外旋60			
3	normal_outreach_90		外展90			
4	normal_in_ro_20_outreach_90	完全正常接骨	内旋20	外展90		
5	normal_ex_ro_20_outreach_90		外旋20	外展90		
6	in_set_20_in_ro_60	内旋20接骨	内旋60			
7	in_set_20_ex_ro_60		外旋60			
8	in_set_20_outreach_90		外展90			
9	ex_set_20_in_ro_60	外旋20接骨	内旋60			
10	ex_set_20_ex_ro_60		外旋60			
11	ex_set_20_outreach_90		外展90			
